# Reversible ratiometric detection of highly reactive hydropersulfides using a FRET-based dual emission fluorescent probe†
†Electronic supplementary information (ESI) available: Full experimental procedures, synthesis and characterization of compounds. See DOI: 10.1039/c6sc03856e



**DOI:** 10.1039/c6sc03856e

**Published:** 2016-09-26

**Authors:** Ryosuke Kawagoe, Ippei Takashima, Shohei Uchinomiya, Akio Ojida

**Affiliations:** a Graduate School of Pharmaceutical Sciences , Kyushu University , 3-1-1 Maidashi, Higashi-ku , Fukuoka , 812-8582 , Japan

## Abstract

Hydropersulfide (R–SSH) is an important class of reactive sulfur species (RSS) involved in a variety of physiological processes in mammals. A fluorescent probe capable of real-time detection of hydropersulfide levels in living cells would be a versatile tool to elucidate its roles in cell signalling and redox homeostasis. In this paper, we report a ratiometric fluorescent probe for hydropersulfide sensing, based on a fluorescence resonance energy transfer (FRET) mechanism. This sensing mechanism involves a nucleophilic reaction of a hydropersulfide with the pyronine-unit of the probe, which modulates the intramolecular FRET efficiency to induce a dual-emission change. The reversible nature of this reaction allows us to detect increases and decreases of hydropersulfide levels in a real-time manner. The probe fluorometrically sensed highly reactive hydropersulfides, such as H_2_S_2_ and Cys-SSH, while the fluorescence response to biologically abundant cysteine and glutathione was negligible. Taking advantage of the reversible and selective sensing properties, this probe was successfully applied to the ratiometric imaging of concentration dynamics of endogenously produced hydropersulfides in living cells.

## Abstract 2

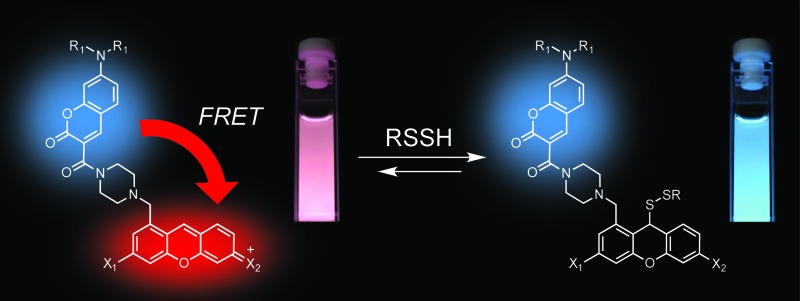
A ratiometric fluorescent probe that can visualize endogenously produced hydropersulfides has been developed.

## Introduction

Reactive sulfur species (RSS) are a family of sulfur-containing molecules endogenously produced in biological systems. Among RSS, hydropersulfides (R–SSHs) such as hydrogen persulfide (H_2_S_2_) and cysteine hydropersulfide (CysSSH) are increasingly recognized as an important class of RSS that modulate a variety of physiological events in mammals. For instance, hydropersulfides are found to play a role in *S*-sulfhydration of Cys residues of proteins as signalling molecules.^1,2^ Previous reports proposed that protein *S*-sulfhydration is mediated by the reaction of hydrogen sulfide (H_2_S) with oxidised cysteine residues such as *S*-sulfenic acid (S–OH) and *S*-nitrosothiol (SNO).^3–7^ However, recent reports suggested that hydropersulfides serve as the main reactive species that directly *S*-sulfhydrate numerous proteins.^8–13^ In particular, it has been revealed that *S*-sulfhydration regulates the functions of important classes of proteins involved in cell redox homeostasis,^8–10^ metabolism,^11^ and signal transduction.^12^ Meanwhile, Akaike suggested that hydropersulfides such as *S*-sulfhydrated glutathione (GSSH) serve as potent reducing agents in redox signalling, and may provide a primary and potent antioxidant defence in cells.^1^

Hydropersulfides (R–SSHs) are generated by different pathways in biological systems. It has been reported that hydrogen persulfide (H_2_S_2_) can be formed by the oxidation of endogenous H_2_S by reactive oxygen species (ROS).^14^ Akaike found that CysSSH is biosynthesized from cystine (CysS-SCys) by two major enzymes: cystathionine β-synthetase (CBS) and cystathionine γ-lyase (CSE).^1,15^ He also proposed that the enzymatically generated CysSSH is converted to GSH-based hydroper-/hydropolysulfides (*e.g.*, GSSH, GSSSH, *etc.*) through persulfide interchange reactions. Meanwhile, Banerjee proposed that sulfide oxidation pathways in mitochondria are the important source of RSS, such as GSSH.^16^ Despite the extensive study of the hydropersulfide formation pathways, regulation of their levels in cells, especially their reducing mechanism, remains largely elusive. Recent reports proposed thioredoxin (Trx) as an important enzyme that reduces CysSSH, although clear evidence for this process has not yet been provided.^17^

**Fig. 1:**
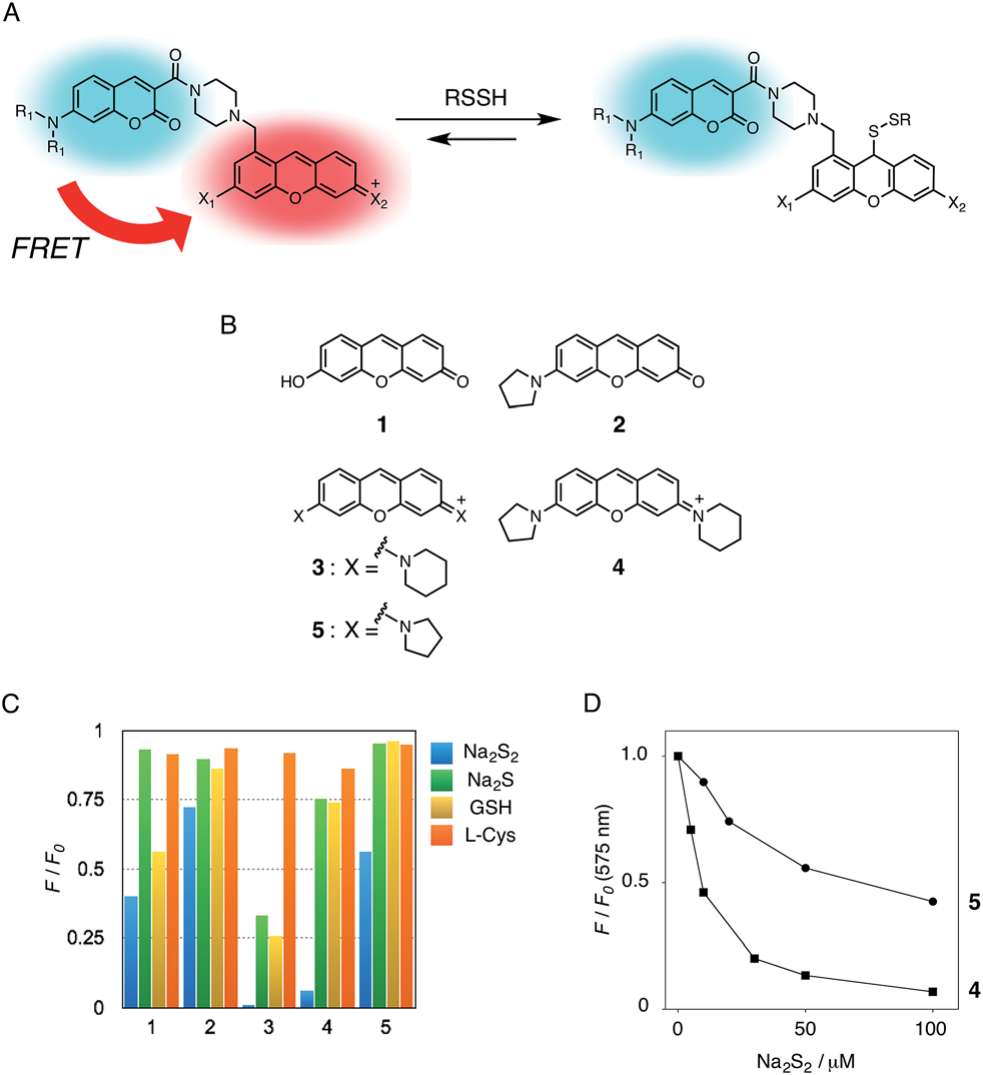
(A) Mechanism of the FRET-based ratiometric fluorescence sensing of hydropersulfide. (B) Structure of the xanthene derivatives. (C) Fluorescence quenching efficiency (*F*/*F*_0_) of **1–5** upon addition of Na_2_S_2_ (50 μM), Na_2_S (50 μM), GSH (5 mM), and l-Cys (1 mM). The data were measured 10 min after addition of the thiol compounds. Measurement conditions: [probe] = 5 μM in 50 mM HEPES, 10 mM NaCl, 1 mM MgSO_4_, pH 7.4, 25 °C. *λ*_ex_ = 480 nm (**1**), 500 nm (**2**), 530 nm (**3**, **4**, **5**). (D) Fluorescence titration profile of **4** (■) and **5** () upon addition of Na_2_S_2_ (0–100 μM). Measurement conditions: [probe] = 5 μM in 50 mM HEPES, 10 mM NaCl, 1 mM MgSO_4_, pH 7.4, 25 °C. *λ*_ex_ = 530 nm.

To understand the varied roles of hydropersulfides in biological systems, it is critical to develop a new analytical tool that allows us to detect the formation and consumption of these RSS species. Fluorescent probes which are available for real-time cell imaging could meet this requirement.^18–23^ In this regard, Xian *et al.* recently reported a series of selective fluorescent probes for hydroper-/hydropolysulfides (H_2_S_*n*_, *n* > 1).^18–20^ They ingeniously exploited the high nucleophilic activity of H_2_S_*n*_ to develop reaction-based turn-on fluorescence probes, which were successfully applied to the visualization of intracellular H_2_S_*n*_. However, due to the irreversible nature of the reactions, it was intrinsically difficult to monitor reversible concentration dynamics of intracellular H_2_S_*n*_ using these probes. In this paper, we report the development of a ratiometric fluorescent probe for detecting hydropersulfides, based on intramolecular fluorescence resonance energy transfer (FRET) (Fig. 1A).^24^ The sensing mechanism of this probe involves a reversible nucleophilic attack of a highly reactive hydropersulfide species on the pyronine fluorophore. This adduct formation disrupts the conjugation structure of the xanthene ring, decreasing the intramolecular FRET efficiency due to a change in the spectral overlap between the coumarin fluorescence (FRET donor) and the xanthene absorbance (FRET acceptor), which causes a clear dual-emission signal change. Taking advantage of this reversible sensing property, the probe was successfully applied to detect the concentration dynamics of hydropersulfides in living cells, demonstrating the utility of the probe as a chemical tool in RSS research.

## Results and discussion

### Molecular design of the probe

In the previous study, we reported that the fluorescence of the xanthene derivative **1** significantly decreased upon addition of a large excess of glutathione (GSH, ∼10 mM) under neutral aqueous conditions.^25^ The spectroscopic analyses revealed that xanthene **1**, which lacks a C9 aromatic substituent unlike fluorescein, was susceptible to nucleophilic attack by thiol species and readily converted to a non-fluorescent adduct. Since hydropersulfides (RSSHs) are more nucleophilic than stable thiols such as GSH and H_2_S,^26^ we thought that this reaction-based fluorescence quenching could be exploited for selective detection of hydropersulfides. As an initial attempt, we synthesized a series of xanthene derivatives bearing the different substituents (Fig. 1B), and evaluated their fluorescence responses toward several biological thiol species. The results are summarized in Fig. 1C and S1.† Compound **1** showed a marked decrease in fluorescence (*F*/*F*_0_ = 40%) upon treatment with 50 μM sodium disulfide (Na_2_S_2_), the extent of which is much larger than that induced by addition of the same concentration of Na_2_S (*F*/*F*_0_ = 93%) and a high concentration (1 mM) of cysteine (*F*/*F*_0_ = 91%). However, **1** also responded to a biologically relevant concentration of GSH (5 mM) with a high quenching efficiency (*F*/*F*_0_ = 56%), indicative of the low selectivity of **1** among biologically relevant thiols. The rhodol-type compound **2** exhibited a rather non-selective weak fluorescence response to the thiol species. The pyronine-type compound **3**, possessing two six-membered piperidine rings, is highly susceptible to thiol species with significant fluorescence quenching efficiencies (*F*/*F*_0_ < 30%), except for l-cysteine. However, pyronines **4** and **5**, which possess one and two five-membered pyrrolidine rings, respectively, showed selective fluorescence responses (*F*/*F*_0_ = 6% and 56%, respectively) toward Na_2_S_2_ (50 μM). The formation of the H_2_S_2_ adduct with **5** was confirmed by a ^1^H-NMR experiment (Fig. S2†). The fluorescence response of **4** and **5** toward Na_2_S_2_ was further evaluated by titration with different concentrations of Na_2_S_2_. As shown in Fig. 1D and S3,† **4** was more sensitive than **5** and showed a substantial decrease in fluorescence with a low concentration of Na_2_S_2_ (below 10 μM). The varied fluorescence response of these pyronine-type probes, depending on the substituents, would be reasonably explained by the different electron donating abilities of the cyclic amines. That is, the five-membered pyrrolidine can act as a stronger electron donating substituent than the six-membered piperidine,^25^ so that the tolerance to nucleophilic attack by Na_2_S_2_ is in the order of **5** > **4** > **3**.

**Fig. 2:**
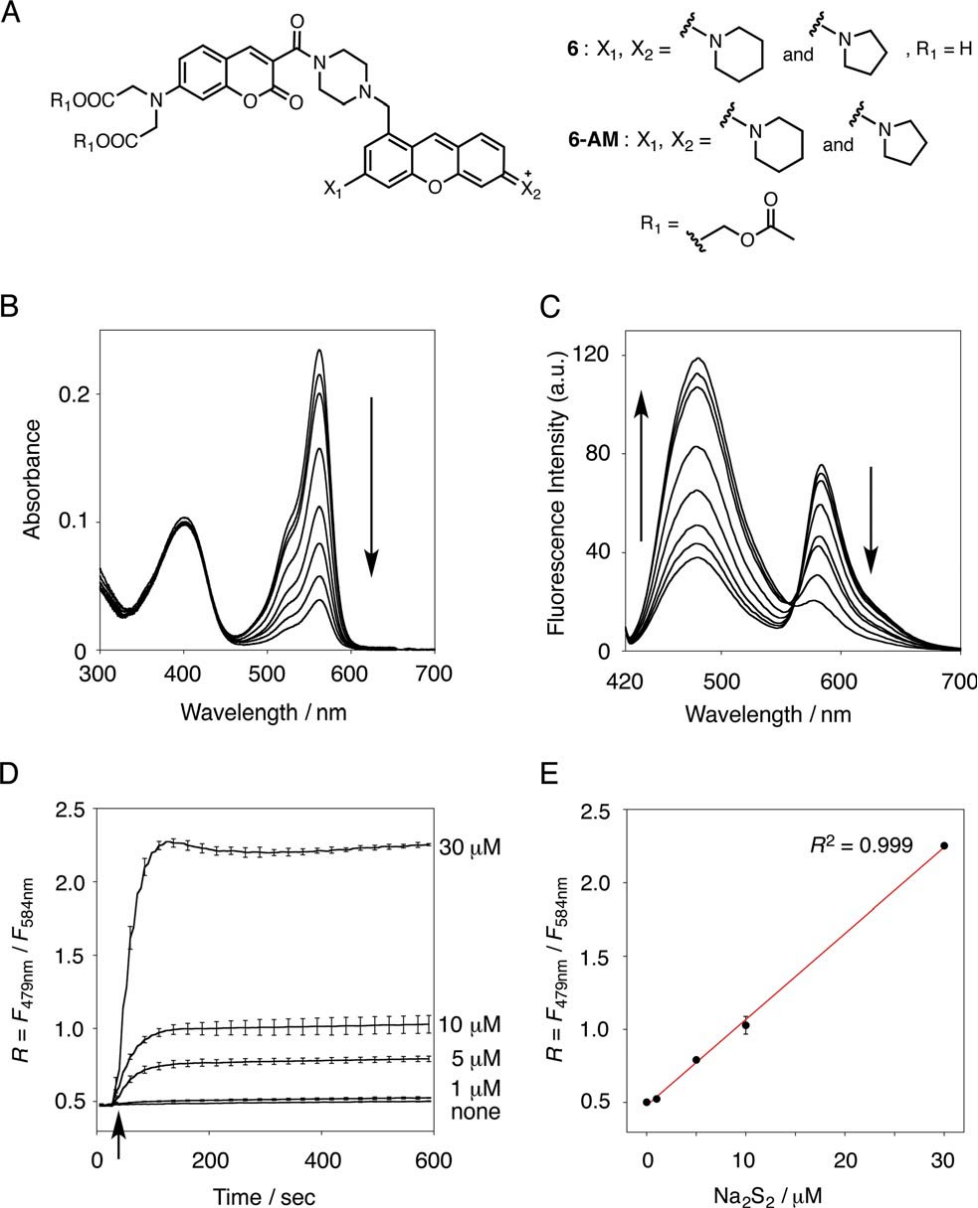
(A) Structures of probe **6** and **6**-AM. (B and C) Absorption and fluorescence spectral changes of **6** (5 μM) upon addition of Na_2_S_2_ (0–100 μM). Measurement conditions: [**6**] = 5 μM in 50 mM HEPES, 10 mM NaCl, 1 mM MgSO_4_, 0.4% Tween, pH 7.4, 25 °C. *λ*_ex_ = 410 nm. (D) Time-dependent change of the ratio value (*R* = *F*_479 nm_/*F*_584 nm_) of **6** (5 μM) upon addition of Na_2_S_2_ (0–30 μM). Na_2_S_2_ was added at 30 s, as indicated by the arrow. Measurement conditions: [**6**] = 5 μM in 50 mM HEPES, 10 mM NaCl, 1 mM MgSO_4_, 0.4% Tween, pH 7.4, 25 °C. *λ*_ex_ = 410 nm. *n* = 3. (E) Plot of the ratio value *R* (*F*_479 nm_/*F*_584 nm_) 600 s after addition of Na_2_S_2_ (0–30 μM).

**Scheme 1:**
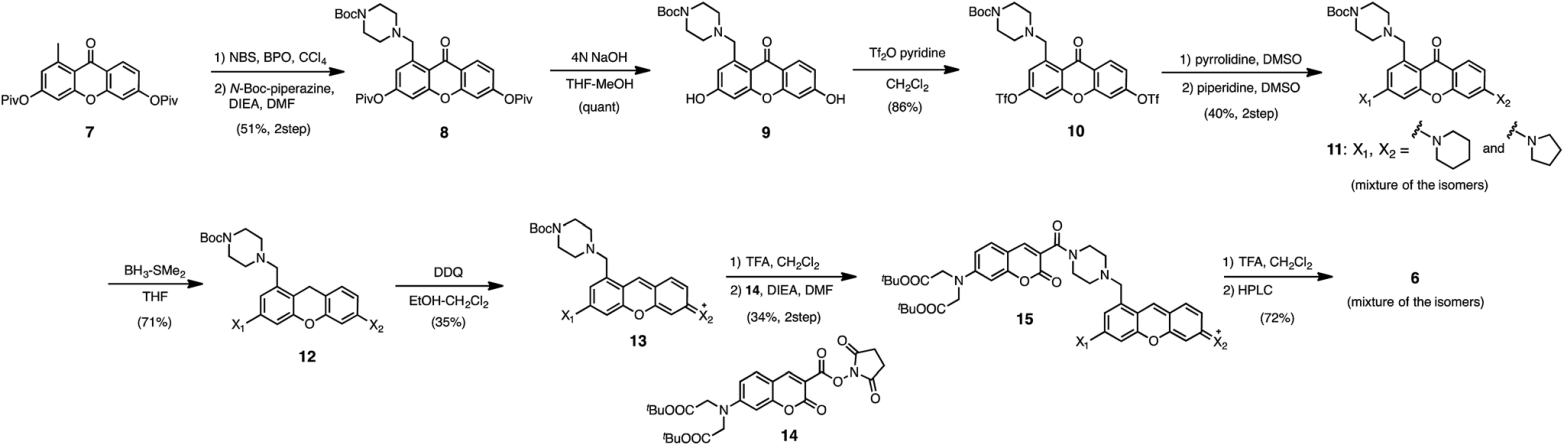
Synthesis of probe **6**.

We selected pyronine **4** as a fluorescent subunit of the ratiometric probe for hydropersulfides, on account of its selective and sensitive detection of Na_2_S_2_, as shown in Fig. 1. The structure of the newly designed dual-emission probe **6** is shown in Fig. 2A. The probe possesses a coumarin as the FRET donor, which is conjugated to a pyronine unit as the FRET acceptor through a rigid linker. The two carboxylate groups are introduced into the coumarin unit in order to increase the hydrophilicity of the probe, which prevents its leakage from cells during imaging experiments. The synthesis of probe **6** is shown in Scheme 1. The radical bromination of **7** with *N*-bromosuccinimide (NBS) and the subsequent nucleophilic reaction with *N*-Boc-piperazine yielded **8**. After the deprotection, **8** was converted to bis-triflate **10**, which was sequentially reacted with pyrrolidine and piperidine to give **11** as a mixture of the substitution isomers. The keto-reduction of **11** with borane-SMe_2_ and the subsequent oxidation using DDQ yielded the pyronine **13**. After the removal of the Boc group, **13** was subjected to a conjugation reaction with the *N*-hydroxysuccinimide ester of coumarin **14** to give **15**. Finally, the deprotection of the *tert*-butyl ester groups of **15** and the following HPLC purification provided **6** as a mixture of the isomers.

### Ratiometric fluorescence sensing of hydropersulfides

The functional analysis of probe **6** was initially conducted in a neutral aqueous solution. A solution of **6** (5 μM) in 50 mM HEPES buffer (pH 7.4) showed two distinct UV peaks at 400 nm and 562 nm (Fig. 2B), which correspond to the absorbance of coumarin and pyronine, respectively. Upon titration with sodium disulfide Na_2_S_2_ (0–100 μM), the absorbance at 562 nm decreased gradually to *ca.* 25% of its original intensity, whereas the absorbance at 400 nm scarcely changed. In the fluorescence spectrum, a solution of **6** (5 μM) showed two distinct emissions at 479 nm and 584 nm due to the coumarin and pyronine units, respectively, when excited at 410 nm (Fig. 2C). This dual-emission spectrum changed dramatically in a see-saw manner upon addition of Na_2_S_2_ (0–100 μM). The large decrease in emission at 584 nm and the concomitant increase in emission at 479 nm strongly suggest that FRET between the coumarin and xanthene units is cancelled as a result of a decrease in the spectral overlap between the coumarin emission and the pyronine absorption (Fig. S4†). The FRET efficiency of **6** was calculated to be 60% in the initial state, which decreased to 31% in the presence of 100 μM Na_2_S_2_. Fig. 2D shows the time-lapse detection of the fluorescence response of **6** toward Na_2_S_2_. The ratio value *R* (*F*_479 nm_/*F*_584 nm_) rapidly increased upon addition of Na_2_S_2_ (0–30 μM) and reached a plateau almost within 60 s. The plot of *R* value against the concentration of Na_2_S_2_ (0–30 μM) shows a linear relationship (Fig. 2E), indicative of the highly quantitative nature of the ratiometric detection of hydropersulfides using **6**. Probe **6** was able to detect as low as 1.0 μM Na_2_S_2_ based on the calculation of the detection limit (3*σ*). This sensitivity is sufficiently high for detection of intracellular hydropersulfides, the concentration of which was reported to be around ten micromolar under basal conditions.^1^

**Fig. 3:**
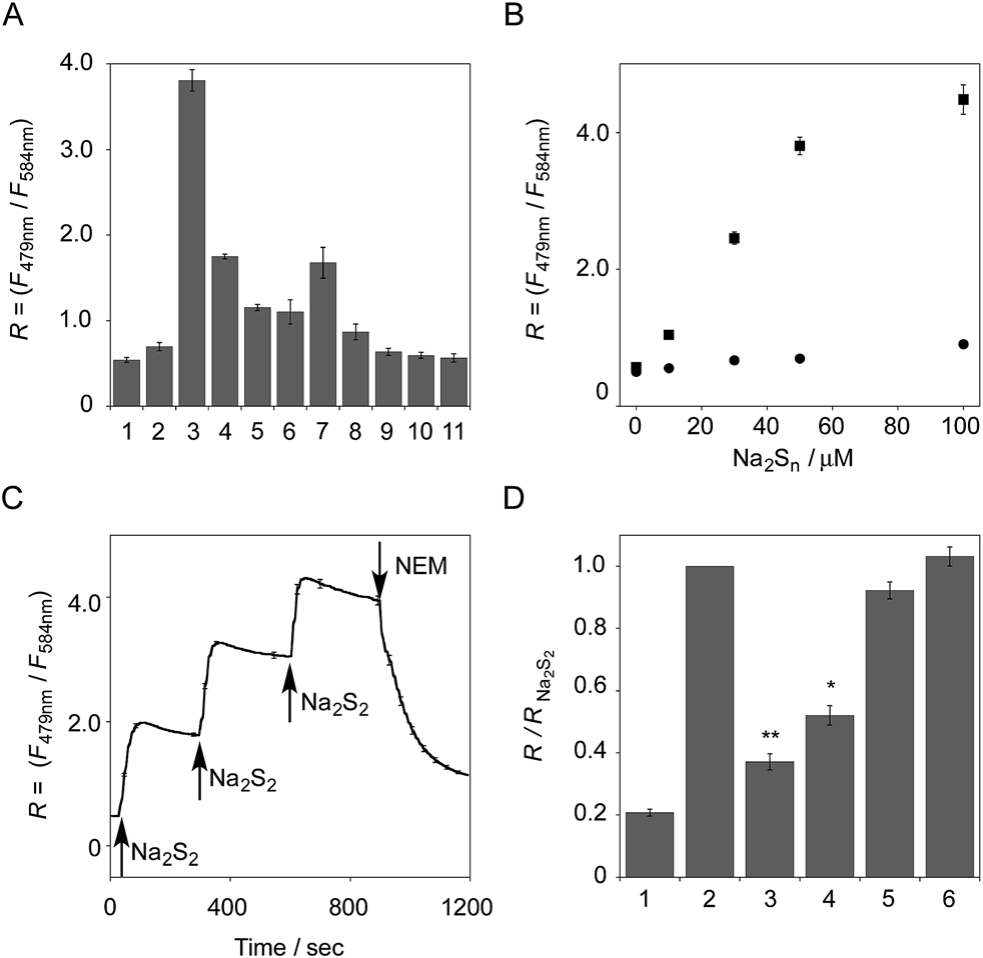
(A) The ratio value (*R* = *F*_479 nm_/*F*_584 nm_) of **6** in the presence of various thiol species: (1) none, (2) Na_2_S (50 μM), (3) Na_2_S_2_ (50 μM), (4) Na_2_S_4_ (50 μM), (5) CysSSH (NOC7 (50 μM) + Na_2_S (50 μM) + l-Cys (50 μM)), (6) GSSH (NOC7 (50 μM) + Na_2_S (50 μM) + GSH (50 μM)), (7) mixture of NaOCl (50 μM) and Na_2_S (50 μM) in 0.1 M NaOH, (8) mixture of NaOCl (50 μM) and Na_2_S (50 μM) in 50 mM HEPES buffer (pH 7.4), (9) GSH (5 mM), (10) l-Cys (1 mM), (11) cystine (0.5 mM). Measurement conditions: [**6**] = 5 μM in 50 mM HEPES, 10 mM NaCl, 1 mM MgSO_4_, 0.4% Tween, pH 7.4, 25 °C. *λ*_ex_ = 410 nm. *n* = 3. (B) Change in the fluorescence intensity ratio of **6** (5 μM) upon addition of Na_2_S_2_ (0–100 μM, ■) and Na_2_S (0–100 μM, ). *n* = 3. (C) Time-trace plot of the ratio value (*R* = *F*_479 nm_/*F*_584 nm_) of **6** (5 μM) upon addition of Na_2_S_2_ (30 μM at 30, 300, and 600 s) and *N*-ethylmaleimide (NEM, 500 μM at 900 s), *n* = 3. (D) The reverse change of the ratio value (*R* = *F*_479 nm_/*F*_584 nm_) of **6** induced by the reactive species. Each bar represents *R* value of: (1) **6** (5 μM), (2) the hydropersulfide adduct of **6** (5 μM) with Na_2_S_2_ (50 μM), (3) the adduct + NaOCl (100 μM), (4) the adduct + NEM (200 μM), (5) the adduct + GSH (5 mM), and (6) the adduct + l-Cys (1 mM). Measurement conditions: 50 mM HEPES, 10 mM NaCl, 1 mM MgSO_4_, 0.4% Tween, pH 7.4, 25 °C. *λ*_ex_ = 410 nm. *n* = 3; *P < 0.05, **P < 0.01 *vs.* hydropersulfide adduct of **6** with Na_2_S_2_ (lane 2).

The sensing selectivity of **6** for various biologically relevant thiol species was evaluated (Fig. 3A). In contrast to the large increase in *R* value induced by Na_2_S_2_ (lane 3), **6** showed a negligible fluorescence response upon addition of the same concentration of Na_2_S (lane 2). This sensing selectivity is reasonably ascribed to the lower p*K*_a_ value of H_2_S_2_ (p*K*_a_ = 5.0) compared to that of H_2_S (p*K*_a_ = 6.9), rendering H_2_S_2_ highly nucleophilic as a thiolate anion (HS_2_^–^) under neutral conditions (pH = 7.4) (Fig. 3B).^26^ A moderate *R* value change was observed upon addition of hydropolysulfide Na_2_S_4_ (lane 4), though this change was apparently smaller than that induced by Na_2_S_2_ (lane 3). The weak fluorescence response of **6** for Na_2_S_4_ might be ascribed to the rather poor nucleophilic activity of the HS_4_^–^ anion due to its extremely low p*K*_a_ value (p*K*_a_ = 3.8).^26^ Probe **6** also showed a moderate increase in the *R* value upon treatment with CysSSH (lane 5) and GSSH (lane 6), which were generated *in situ* from Cys and GSH, respectively, by the reaction with Na_2_S and NO donor (NOC7).^1^ In a similar manner, the mixture of Na_2_S (50 μM) and NaOCl (50 μM) in a basic solution (0.1 M NaOH), which generates Na_2_S_2_ *in situ*, induced a moderate increase of *R* value (lane 7), while their mixture in a neutral solution (50 mM HEPES, pH 7.4), which mainly produces Na_2_S_4_ and Na_2_S_5_,^14^ resulted in a small increase in the *R* value (lane 8). This difference in signal change is consistent with the results obtained by the direct titration with these thiols (lanes 3 and 4). Probe **6** showed a negligible *R* value change upon addition of biologically relevant concentrations of GSH (5 mM, lane 9), l-cysteine (1 mM, lane 10), and cystine (0.5 mM, lane 11). It is noteworthy that the sensing selectivity of **6** for Na_2_S_2_ is apparently higher than that of **5** among these thiol species (Fig. 1C), probably due to steric hindrance and/or the electron configuration effect of the coumarin unit conjugated to the pyronine unit of **6**. Therefore, **6** was able to detect Na_2_S_2_ with a sufficient sensitivity (detection limit = 4.4 μM) even in the presence of 5 mM of GSH (Fig. S5†). We also confirmed that **6** scarcely responded to various redox-relevant compounds, including ROS such as NaOCl and H_2_O_2_, reactive nitrogen species (NOS), ascorbic acid, and KCN (Fig. S6†).

All of these data suggest that probe **6** primarily serves as a selective fluorescent probe for hydropersulfides. It was confirmed that **6** did not show a significant ratio change over the physiological pH range (5.0 to 8.5; Fig. S7†).

The fluorescence response of **6** towards a change in the hydropersulfide level was examined. As shown in Fig. 3C, the *R* value of **6** (5 μM) increased stepwise upon the repeated addition of 30 μM Na_2_S_2_, and reached a plateau almost within 60 s. The subsequent addition of *N*-ethylmaleimide (NEM, 500 μM) to consume Na_2_S_2_ induced a large decrease in the *R* value. These data clearly indicate that **6** can reversibly change its *R* value depending on the concentration of hydropersulfide according to the binding equilibrium shown in Fig. 1A. The reverse fluorescence response of **6** was further evaluated upon addition of other reactive species (Fig. 3D). Addition of NaOCl (100 μM) to the mixed solution of **6** (5 μM) and Na_2_S_2_ (50 μM) also induced a significant decrease in the *R* value (lane 3), as observed with NEM (lane 4). This change is reasonably ascribed to the decrease in Na_2_S_2_ level due to the formation of oxidized sulfurs and hydropolysulfides (H_2_S_*n*_, *n* > 2). Conversely, a small change in the *R* value was induced upon addition of GSH (5 mM, lane 5) and l-Cys (1 mM, lane 6), suggesting that these biologically abundant thiols do not interfere with the hydropersulfide sensing of **6**.

### Ratio imaging of hydropersulfides in living cells

**Fig. 4:**
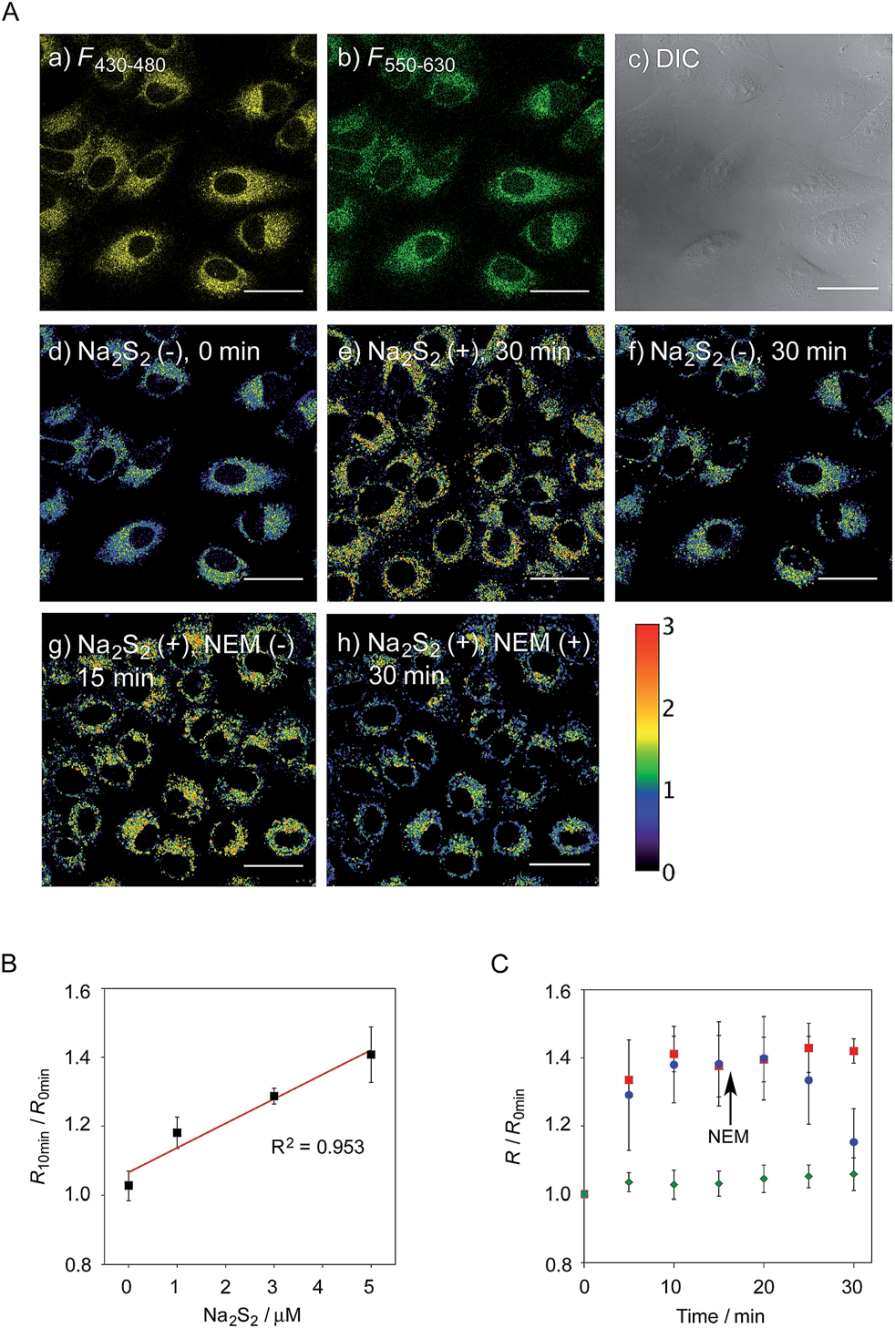
(A) Fluorescence images of A549 cells treated with **6**-AM (5 μM). (a) *F*_430–480_, (b) *F*_550–630_, (c) DIC, and (d) ratio image (*R* = *F*_430–480_/*F*_550–630_). (e and f) Ratio image after 30 min in the presence and absence of Na_2_S_2_ (5 μM), (g and h) ratio image after 15 min in the presence Na_2_S_2_ (5 μM) and subsequent treatment with NEM (100 μM) for 15 min. Scale bar: 30 μm. (B) Ratio value change of A549 cells upon the addition of various concentrations of Na_2_S_2_ (0–5 μM), *n* = 6. (C) Time trace plot of the ratio value in A549 cells upon the treatment with Na_2_S_2_ (5 μM) (red square, *n* = 6), Na_2_S_2_ (5 μM) and the subsequent addition of NEM (100 μM) at 15 min (blue circle, *n* = 4), without Na_2_S_2_ and NEM (green diamond, *n* = 6).

We next applied probe **6** to the ratiometric fluorescence imaging of hydropersulfides (R–SSH) in living cells. For cell imaging, **6** was chemically modified with acetoxymethyl (AM) groups to enhance its membrane permeability.^27^ It was expected that the AM-modified probe, **6**-AM (Fig. 2A), could be readily hydrolyzed by intracellular esterases to liberate **6**, which is unlikely to leak from cells due to its highly polar character. When A549 cells were treated with **6**-AM (5 μM) for 20 min, bright fluorescence of the probe was observed in the cytosolic region of the cells (Fig. 4A(a–d)). A cell viability assay revealed that **6**-AM showed low cytotoxicity to A549 cells at a concentration below 25 μM (Fig. S8†). Addition of 5 μM Na_2_S_2_ to the cell medium induced an obvious change in the fluorescence ratio (*R* = 430–480 nm/550–630 nm) (Fig. 4A(e)) of cells, relative to that observed in the control experiment without Na_2_S_2_ treatment (Fig. 4A(f)). Titration of the **6**-AM pre-stained cells with Na_2_S_2_ (0–5 μM) showed a linear dependence between Na_2_S_2_ concentration and *R* value (Fig. 4B and S9†), demonstrating the accurate hydropersulfide sensing property of **6** in living cells. The time-lapse imaging revealed that the *R* value increased immediately after the addition of 5 μM Na_2_S_2_ and reached a steady state (*R* = ∼1.4) after 10 min (Fig. 4C). The subsequent treatment of the cells with 100 μM *N*-ethylmaleimide (NEM) induced a significant decrease in the *R* value (Fig. 4A(g, h) and C) due to the decrease of the hydropersulfide level by the nucleophilic reaction with NEM, demonstrating the reversible sensing property of **6** in living cells.

**Fig. 5:**
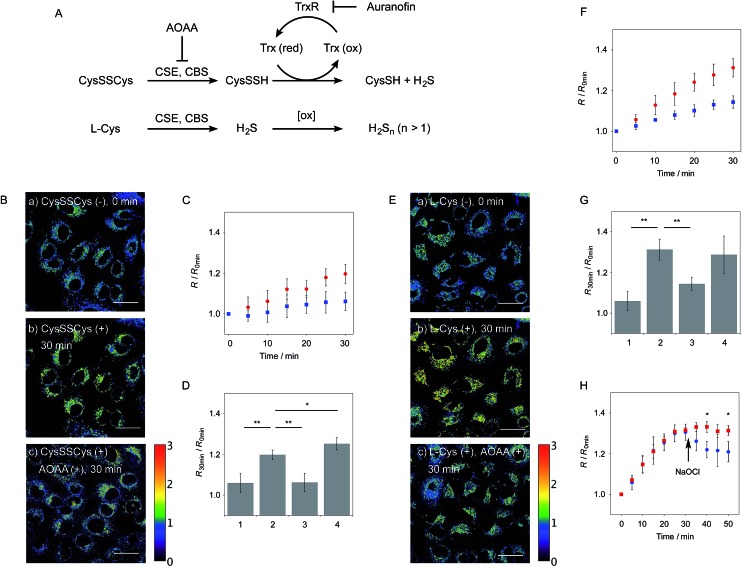
Ratiometric detection of endogenously produced hydropersulfide in A549 cells. (A) Production and degradation pathways of hydropersulfides in cells. (B) Ratio images of A549 cells treated with **6**-AM (5 μM) (a) before addition of cystine, (b) 30 min after addition of cystine (200 μM), (c) 30 min after addition of cystine (200 μM) in the presence of AOAA (1 mM). (C) Time trace plot of the ratio value (*R* = *F*_430–480_/*F*_550–630_) change in A549 cells upon treatment with cystine (200 μM) in the absence (red circle, *n* = 6) and presence of AOAA (1 mM) (blue square, *n* = 6). (D) Comparison of the ratio value *R* change in A549 cells upon treatment with cysteine for 30 min: (1) control (without cystine), *n* = 6, (2) cystine (200 μM), *n* = 6, (3) cystine (200 μM) in the presence of AOAA (1 mM), *n* = 4, (4) cystine (200 μM) in the presence of auranofin (2 μM), *n* = 3. (E) Ratio images of A549 cells treated with **6**-AM (5 μM) (a) before addition of l-cysteine, (b) 30 min after addition of l-cysteine (200 μM), (c) 30 min after addition of l-cystine (200 μM) in the presence of AOAA (1 mM). (F) Time trace plot of the ratio value *R* change in A549 cells upon treatment with l-cysteine (200 μM) in the absence (red circle, *n* = 3) and presence of AOAA (1 mM) (blue square, *n* = 3). (G) Comparison of the ratio value *R* change in A549 cells upon treatment with l-cysteine for 30 min; (1) control (without cysteine), *n* = 6, (2) l-cysteine (200 μM), *n* = 4, (3) l-cysteine (200 μM) in the presence of AOAA (1 mM), *n* = 3, (4) l-cysteine (200 μM) in the presence of auranofin (2 μM), *n* = 5. (H) Time trace plot of the ratio value *R* change in A549 cells upon treatment with l-cysteine (200 μM) (red square, *n* = 3) and upon treatment with l-cysteine (200 μM) and NaOCl (300 μM) (blue circle, *n* = 3). NaOCl was added 15 min after addition of l-cysteine. Conditions: *λ*_ex_ = 405 nm, *R* = *F*_430–480_/*F*_550–630_ nm. Scale bar: 30 μm. *P < 0.05, **P < 0.01.

The probe **6**-AM was further applied to the ratiometric detection of endogenous hydropersulfides produced by enzymes in living cells. It has been reported that CSE and CBS are the major enzymes responsible for generation of cysteine hydropersulfide (CysSSH) from cystine (CysSSCys) in A549 cells.^1^ When A549 cells, pre-stained with **6**-AM, were treated with cystine (CysSSCys), a gradual increase in the *R* value was observed (Fig. 5B and C). This change was effectively suppressed on treatment of the cells with aminooxyacetic acid (AOAA), an inhibitor of CSE and CBS (Fig. 5B and D, lane 3).^28^ Conversely, treatment of the cells with auranofin, an inhibitor for thioredoxin reductase (TrxR), induced a statistically significant increase in the *R* value (Fig. 5D, lane 4 and S10†), suggesting an increase in the intracellular hydropersulfide level as a result of the inhibition of Trx activation by TrxR (Fig. 5A). This result is consistent with the recent report that Trx catalyses reduction of Cys-SH as a major regulator of its intracellular level.^17^ It has been reported that hydropersulfide is also produced in living cells by the oxidation of H_2_S, which is generated from l-Cys by CSE and CBS.^20,22^ To confirm this point, fluorescence imaging of A549 cells treated with l-Cys (200 μM) was performed using **6**-AM. As shown in Fig. 5E and F, the *R* value largely increased in a time-dependent manner in the living cells. Treatment of the cells with AOAA effectively suppressed the increase in *R* value induced by l-Cys (Fig. 5E and G, lane 3). Unlike the case of the cysteine experiment (Fig. 5D), inhibition of TrxR by auranofin did not cause an increase in the *R* value (Fig. 5G, lane 4 and S11†), implying that the direct conversion of l-Cys to CysSSH is not a major pathway of the hydropersulfide formation in living cells. All of these data suggest that hydropersulfides are generated from l-Cys through the enzyme-mediated H_2_S formation and subsequent oxidation in living cells. Finally, the addition of high concentration NaOCl (300 μM) induced a significant decrease in the *R* value in cells (Fig. 5H and S12†), indicating that the decrease in hydropersulfide level was a result of its oxidative degradation to oxidized sulfurs and hydrogen polysulfides (H_2_S_*n*_, *n* > 2).

## Conclusion

In conclusion, we have developed a ratiometric fluorescent probe **6** that can visualize the endogenously produced hydropersulfides in living cells. To our knowledge, probe **6** is the first example of a ratiometric fluorescent probe that can reversibly detect intracellular hydropersulfide levels. Since the research field of reactive sulfur species (RSS) is still in its infancy, probe **6** would serve as a versatile analytical tool, not only for understanding the chemical nature of hydropersulfide in biological systems, but also for elucidating the roles of hydropersulfide in cell signalling and redox homeostasis. For this purpose, further functional improvements in probe **6**, which include sensing selectivity for a single hydropolysulfide species and/or localizability to a certain cell compartment, would be desirable to realize more precise and quantitative analysis of RSS. Research along these lines is currently underway in our laboratory.

## Experimental section

### Synthesis and characterization of the compounds

The syntheses and characterization of probes **1–6** and **6**-AM are described in the ESI.†

### Fluorescence measurement

Fluorescence titration was conducted with a solution (3 mL or 0.5 μL) of the probe in a quartz cell. Typically, a freshly prepared aqueous stock solution of Na_2_S_2_ was added to a solution of **6** (5 μM) in 50 mM HEPES, 10 mM NaCl, 1 mM MgSO_4_, pH 7.4 with 0.4% Tween at 25 °C and the fluorescence emission spectra were measured after 10 min (*λ*_ex_ = 410 nm) with a Perkin Elmer LS55 fluorescence spectrometer.

### Cell culture

A549 cells were cultured in high glucose Dulbecco's Modified Eagle's Medium (DMEM, Gibco) supplemented with 10% fetal bovine serum (FBS, Gibco) and 1% antibiotic-antimycotic solution (Gibco) at 37 °C under a humidified atmosphere of 5% CO_2_ in air. A subculture was performed every 3–4 days from subconfluent (<80%) cultures using a trypsin–EDTA solution. For the fluorescence bioimaging, cells were cultured for 2 days in a 35 mm glass-bottomed dish (Iwaki Scitech).

### Fluorescence imaging of exogenous H_2_S_2_ in A549 cells

Fluorescence imaging was conducted with a confocal laser scanning microscope (LSM 780, Zeiss) equipped with a 63× objective lens. The following detection channels were chosen for the ratiometric imaging; Ch1 *λ*_ex_ = 405 nm, *λ*_em_ = 430–480 nm, and Ch2 *λ*_ex_ = 405 nm, *λ*_em_ = 550–630 nm. In a glass-based dish, A549 cells in HBS buffer (107 mM NaCl, 6 mM KCl, 1.2 mM MgSO_4_, 2.0 mM CaCl_2_, 11.5 mM glucose, 20 mM HEPES, pH 7.4) were incubated with **6**-AM (5 μM) for 20 min at 37 °C under a humidified atmosphere of 5% CO_2_ in air. After removal of excess probe and washing with HBS buffer, the cells were treated with Na_2_S_2_ (0–5 μM, final conc.) and subjected to the fluorescence imaging. For the imaging of the endogenously produced hydropersulfides, A549 cells, pre-stained with **6**-AM (5 μM), were treated with cystine (200 μM) or l-cysteine (200 μM). For inhibition of CSE and CBS enzymes, the cells were pre-treated with AOAA (aminooxyacetic acid, 1 mM, Sigma) in HBS buffer for 1 h before staining with **6**-AM. For inhibition of TrxR, the cells were pre-treated with auranofin (2 μM, Wako) in HBS buffer for 1 h before staining with **6**-AM.
