# Supplementary material for: Reversible ratiometric detection of highly reactive hydropersulfides using a FRET-based dual emission fluorescent probe
Source: Chem Sci. 2016 Sep 26;8(2):1134–40. doi: 10.1039/c6sc03856e (PMC5369533; doi:10.1039/c6sc03856e)
Supplement: Supplementary file 1 [file SC-008-C6SC03856E-s001.pdf]

Electronic Supplementary Information for

Reversible Ratiometric Detection of Highly Reactive Hydropersulfides Using a FRET-Based Dual Emission Fluorescent Probe

Ryosuke Kawagoe,[a] Ippei Takashima,[a] Shohei Uchinomiya,[a] Akio Ojida[a,*]

[a]Graduate School of Pharmaceutical Sciences, Kyushu University, 3-1-1, Maidashi, Higashi-ku, Fukuoka, 812-8582, Japan.

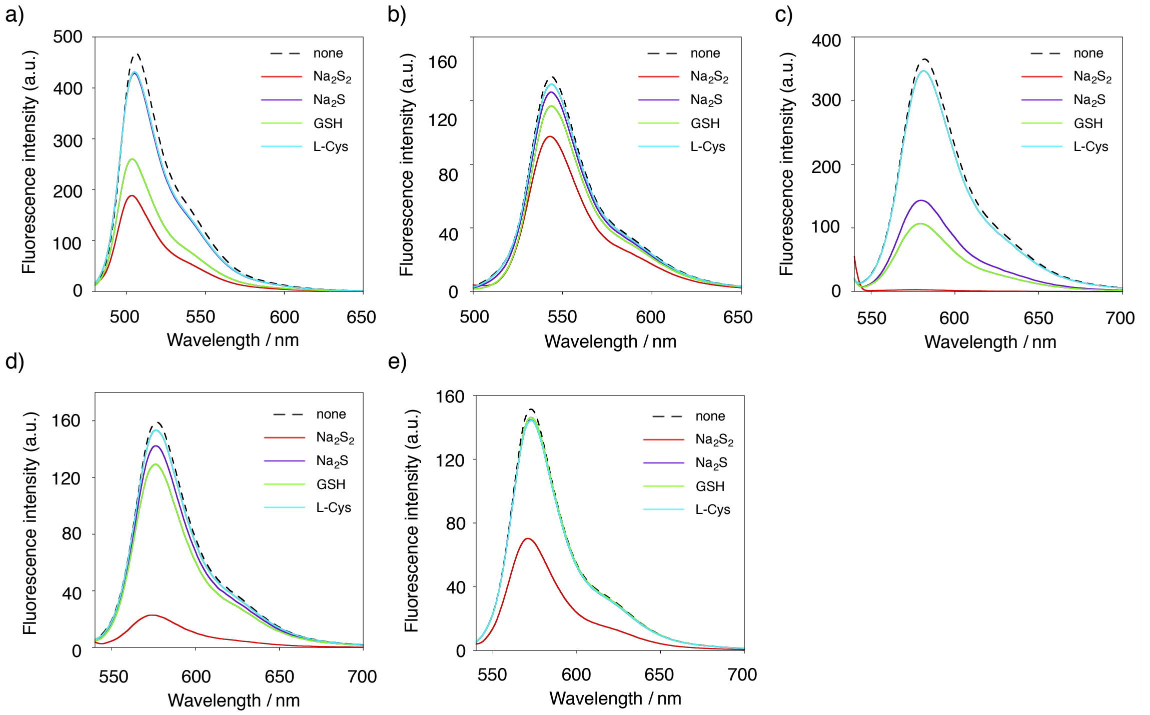

Figure S1. Fluorescence spectral change of **1** (a), **2** (b), **3** (c), **4** (d), and **5** (e) upon treatment with Na_2S_2 (50 μM), Na_2S (50 μM), GSH (5 mM), and L-Cys (1 mM). All data were measured 10 min after addition of the thiol species. Measurement conditions: [probe] = 5 μM in 50 mM HEPES, 10 mM NaCl, 1 mM $MgSO_4$, pH 7.4, 25 °C. λ_{ex} = 480 nm (**1**), 500 nm (**2**), 530 nm (**3**, **4**, **5**).

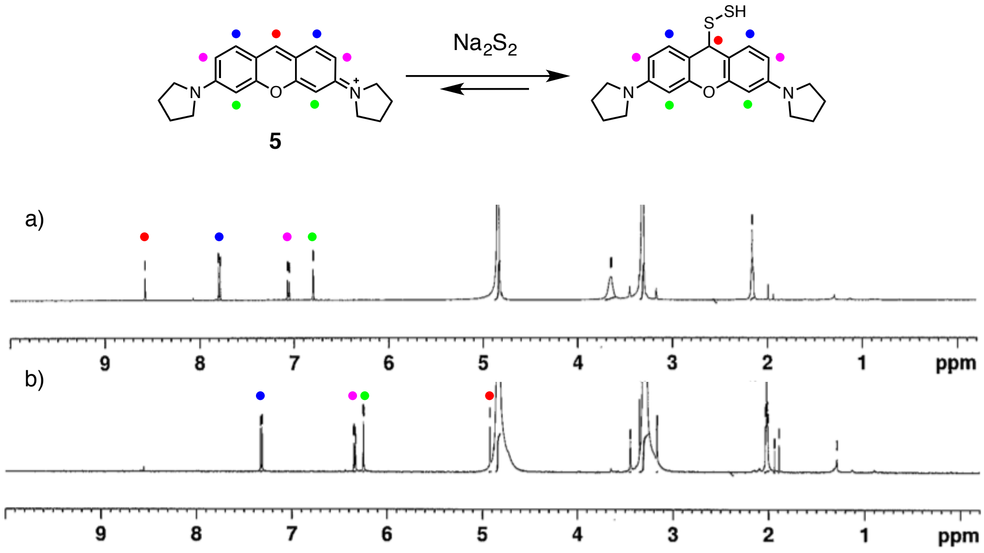

Figure S2. ^{1}H-NMR spectra (500 MHz) of (a) **5** (2 mM) and (b) **5** (2 mM) + Na_2S_2 (10 mM) in CD_3OD.

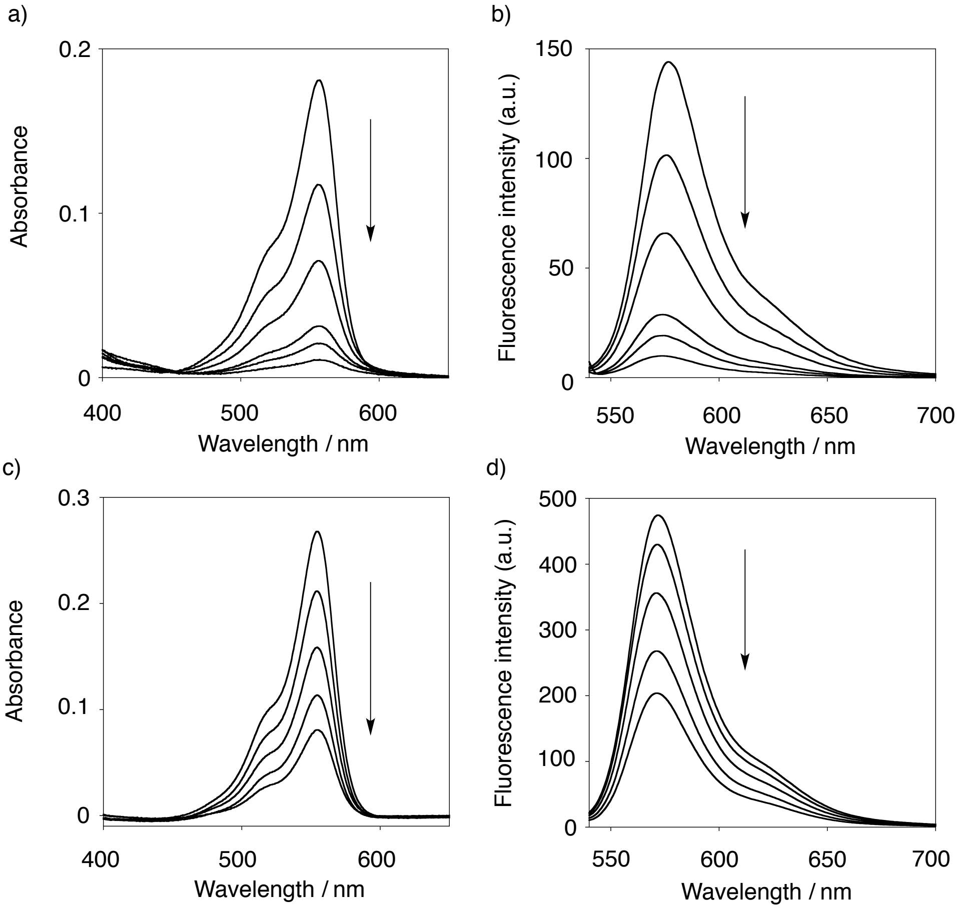

Figure S3. (a, b) Absorbance and fluorescence spectral change of **4** upon addition of Na_2S_2 (0-100 μM). (b, c) Absorbance and fluorescence spectral change of **5** upon addition of Na_2S_2 (0-100 μM). The spectra were measured 5 min after addition of Na_2S_2. Measurement conditions: [probe] = 5 μM in 50 mM HEPES, 10 mM NaCl, 1 mM $MgSO_4$, pH 7.4, 25 °C. λ_{ex} = 530 nm.

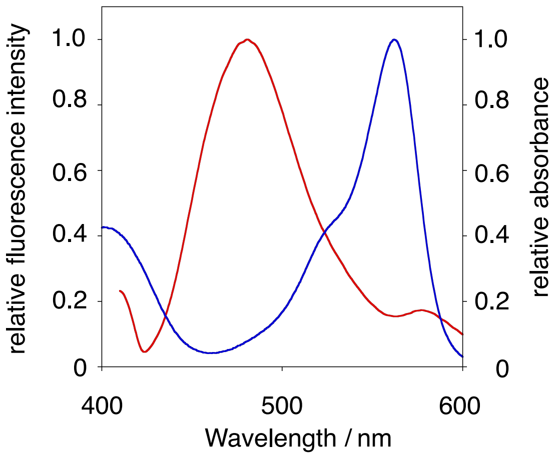

Figure S4. Spectral overlap between the coumarin emission (red line) and the absorption of **6** (blue line). The coumarin mission was measured in the presence of 20 euqiv of Na_2S_2. Each spectrum was normalized by the maximum emission intensity or absorbance. Measurement conditions: 50 mM HEPES, 10 mM NaCl, 1 mM $MgSO_4$ (pH 7.4), 0.4 % Tween, 25 °C, λ_{ex} = 410 nm.

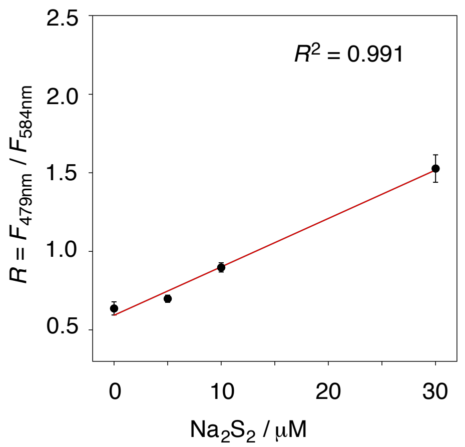

Figure S5. Plot of ratio value R (F_{479nm}/F_{584nm}) of **6** (5 μM) upon addition of Na_2S_2 (0 - 30 μM) in the presence of GSH (5 mM). Measurement conditions: [**6**] = 5 μM, [GSH] = 5 mM in 50 mM HEPES, 10 mM NaCl, 1 mM $MgSO_4$, 0.4 % Tween, pH 7.4, 25 °C. λ_{ex} = 410 nm. n = 3.

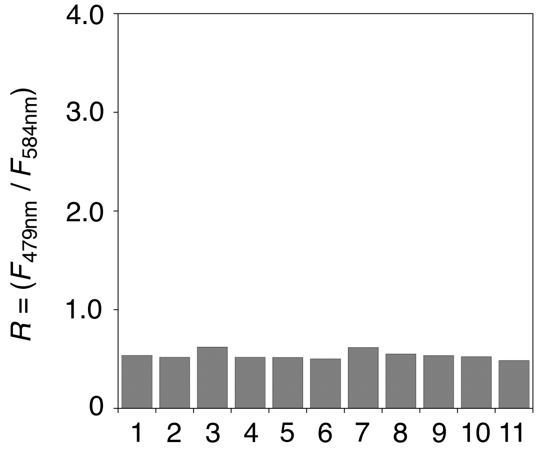

Figure S6. Fluorescence ratio value ($R = F_{479\,nm} / F_{584\,nm}$) of **6** upon treatment with biological reactive species; 1) H_2O_2 (500 μM), 2) ^{t}BuOOH (100 μM), 3) NaClO (50 μM), 4) •OH (100 μM H_2O_2 + 50 μM $Fe(ClO_4)_2$, 5) O2˙⁻(100 μM KO_2), 6) NOC7 (NO donor, 100 μM), 7) ONOO (100 μM), 8) lipoic acid (1 mM), 9) homocysteine (100 μM), 10) ascorbic acid (100 μM), 11) KCN (100 μM). Meaturement conditions: [**6**] = 5 μM, 50 mM HEPES, 10 mM NaCl, 1 mM $MgSO_4$ buffer, 0.4% Tween, pH = 7.4, λ_{ex} = 410 nm.

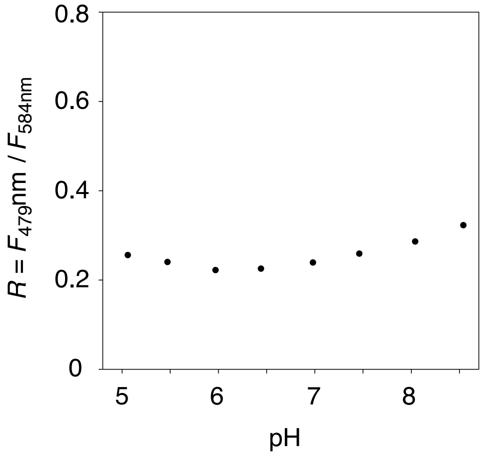

Figure S7. pH-dependency of the ratio value ($R = F_{479\,nm} / F_{584\,nm}$) of **6**. Measurement conditions: [**6**] = 5 μM, 5 mM HEPES + 5 mM CHES + 5 mM MES buffer, 25 °C, λ_{ex} = 410 nm

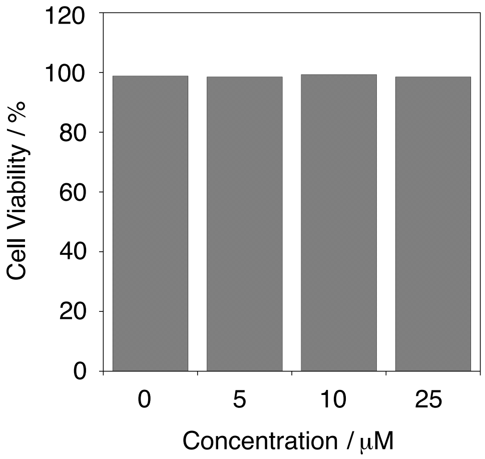

Figure S8. Cell viability test using Trypan blue (0.2 wt%) incubated in HBS buffer for 10 min. The data were obtained from the repetitive (n = 2) experiments. [**6**] = 0, 5, 10, 25 μM incubated in HBS buffer for 2 hr at CO_2 5%, 37 °C

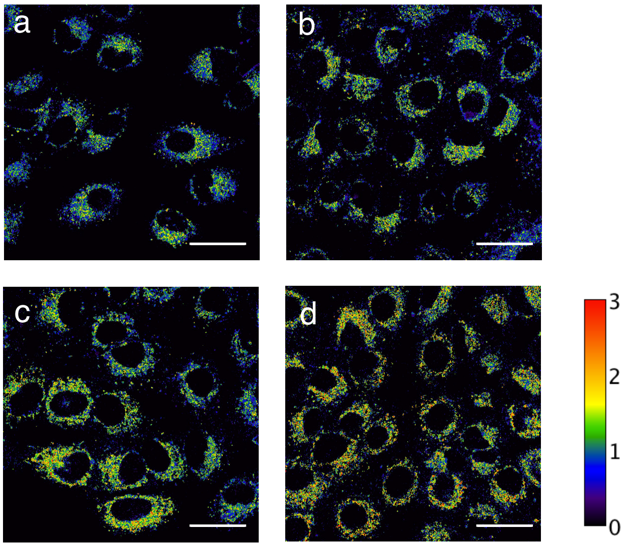

Figure S9. Ratio image of A549 cells pre-treatment with **6** (5 μM) in HBS buffer for 20 min. Ratio image of the cells treated with Na_2S_2 a) none, b) 1 μM, c) 3 μM, d) 5 μM for 10 min. Conditions; λ_{ex} = 405 nm, $R = F_{430\text{-}480} / F_{550\text{-}630}$ nm,. Scale bar: 30 μm.

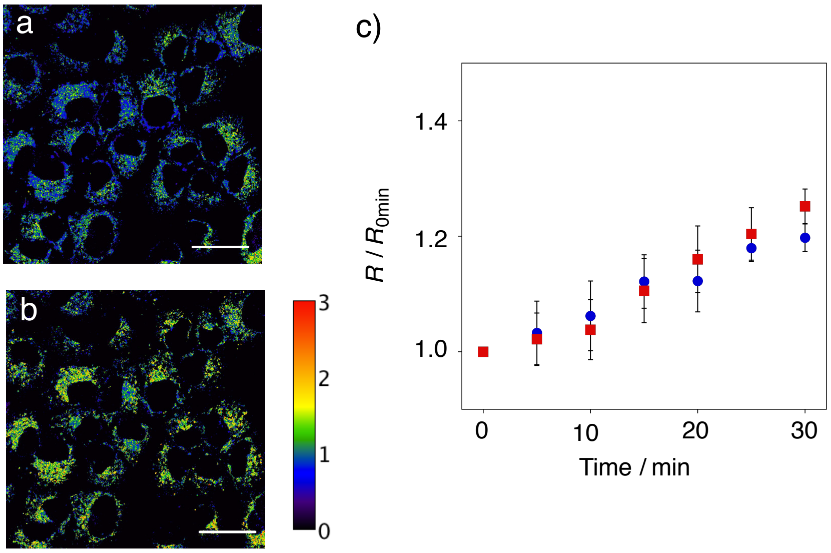

Figure S10. Ratio image of A549 cells stained with **6**-AM (5 μM) a) before addition of cystine, b) 30 min after addition of cystine (200 μM), Conditions; λ_{ex} = 405 nm, $R = F_{430\text{-}480} / F_{550\text{-}630}$ nm,. Scale bar: 30 μm.. c) Time trace plot of the ratio value R change in A549 cells upon treatment with cystine (200 μM, blue circle, n = 6) and cystine (200 μM) in the presence of auranofin (red square, n = 3). For inhibition of TrxR, A549 cells were pre-treated auranofin (2 μM) for 60 min before measurement.

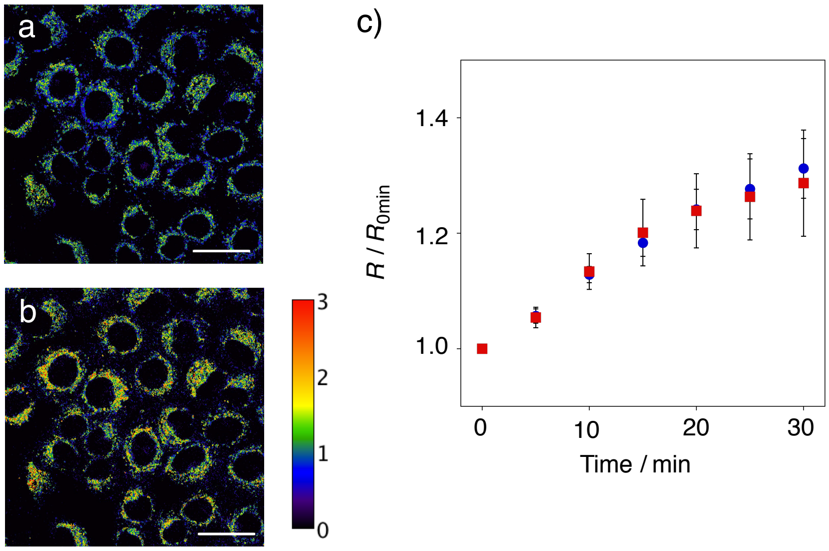

Figure S11. Ratio image of A549 cells stained with **6**-AM (5 μM) a) before addition of L-cysteine, b) 30 min after addition of L-cysteine (200 μM), Conditions; λ_{ex} = 405 nm, $R = F_{430\text{-}480} / F_{550\text{-}630}$ nm,. Scale bar: 30 μm.. c) Time Trace plot of the ratio value R change in A549 cells upon treatment with L-cysteine (200 μM, blue circle, n = 4) and L-cysteine (200 μM) in the presence of auranofin (red square, n = 5). For inhibition of TrxR, A549 cells were pre-treated auranofin (2 μM) for 60 min before measurement.

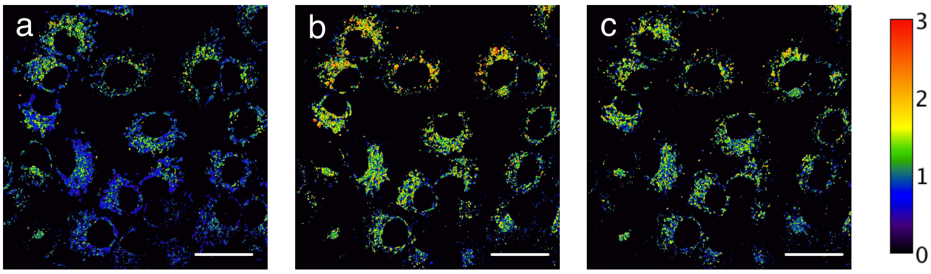

Figure S12. Ratio image of A549 cells stained with **6**-AM (5 μM) a) before addition of L-cysteine, b) 30 min after addition of L-cysteine (200 μM), c) susbsequent treatment with NaOCl (300 μM) for 20 min. Conditions; λ_{ex} = 405 nm, $R = F_{430\text{-}480} / F_{550\text{-}630}$ nm,. Scale bar: 30 μm.

Evaluation of FRET efficiency

FRET efficiency (E) of the **6** and its hydropersulfide adduct was evaluated according to the following equation:

$$E = 1 - \frac{\Phi_D}{\Phi_D^0}$$

Φ_D^0 and Φ_D are the fluorescence quantum yields of the coumarin donor in the presence and absence of the pyronine acceptor, respectively. Φ_D^0 was obtained by the coumarin derivative **46** (0.5 μM) in an aqueous solution (50 mM HEPES, 10 mM NaCl, 1 mM $MgSO_4$ (pH 7.4), 0.4% Tween). Φ_D was obtained by the fluorescence measurement of **6** (1 μM) under the same aqueous conditions in the presence and absence of 100 μM Na_2S_2.

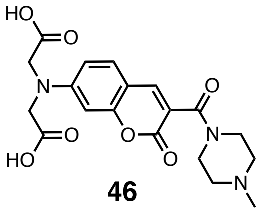

46

Determination of detection limit

Detection limit (DL) of **6** for Na_2S_2 was defined by the following equation, [S1]

$$DL = \frac{[H_2S_2]\, 3\,\sigma}{R_{H2S2} - R_0}$$

, wherein R_{H2S2} and R_0 are the emission ratio value of **6** (F_{479nm} / F_{584nm}, 5 μM) in the presence or absence of a certain concentraion of Na_2S_2 (0 - 30 μM), respectively, and σ is the standard deviation of R. In this concentration range, R value changes in a linear relationship with concentration of H_2S_2. The fluorescent measurement were conducted for 10 min in 50 mM HEPES, 10 mM NaCl, 1 mM MgSO4, 0.4% Tween, pH 7.4 at 25 °C after addition of Na_2S_2. DL in the presence or absence of GSH (5 mM) were calculated to respectively 1.01 μM, 4.41 μM.

General Materials and Methods for Organic Synthesis.

Unless otherwise noted, chemical reagents were purchased from commercial suppliers (Sigma-Aldrich, Tokyo Chemical Industry (TCI), Wako Pure Chemical Industries) and used without further purification. ^{1}H NMR spectra were recorded using a Varian UNITY-400 (400 MHz) and Bruker Ascend 500^{TM} (500 MHz) spectrometer, and chemical shifts (δ, ppm) were referenced to residual solvent peak. ESI mass spectrometry was recorded using a Bruker microTOF II (Bruker Daltonics, USA) spectrometer. HPLC purification was conducted with a HITACHI L-7100 (Hitachi, Japan).

Scheme S1. Synthesis of 6

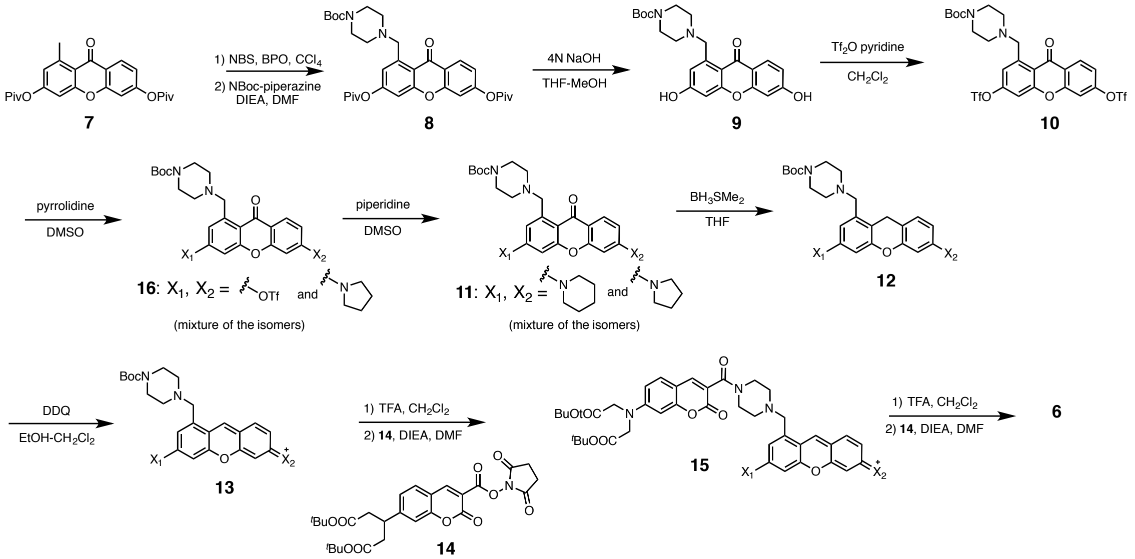

Synthesis of 8

To a solution of **7** (1.05 g, 2.56 mmol) and *N*-bromosuccinimide (0.46 g, 2.56 mmol) in CCl_4 (60 mL) was added a catalytic amount of benzoyl peroxide. The reaction mixture was refluxed for 2 h. After cooled to rt, the solvent was removed in vacuo and the residue was diluted with AcOEt. The organic layers were washed with saturated $NaHCO_3$, water and brine followed by drying over Na_2SO_4. After removal of the solvent in vacuo, the residue was puried by flash column chromatography on silica gel (hexane / AcOEt = 20 /1) to give a crude monobromide (1.80 g) as a yellow oil. To a solution of the crude monobromide (0.87 g, 1.79 mmol) and DIEA (0.75 mL, 5.37 mmol) in dry DMF(10 mL) was added *N*-Boc piperazine (0.67 g, 3.58 mmol) and the solution was stirred at rt for 3 h. After dilution with water, the mixture was extracted with AcOEt. The organic layers were washed with saturated $NaHCO_3$, water and brine. The solvent was dried over Na_2SO_4 and concentrated in vacuo. The residue was purified by

flash column chromatography on silica gel (hexane / AcOEt = 20 /1 → 10 / 1 → 5 / 1) to give **8** (0.78 g, 51% in 2 steps) as a white solid.

^{1}H-NMR (400 MHz, $CDCl_3$) δ 1.39 (9H, s), 1.40 (9H, s), 1.47 (9H, s), 2.57 (4H, s), 3.48 (4H, s). 4.32 (2H, s), 7.06-7.08 (1H, d, J = 8.8 Hz), 7.15 (1H, s), 7.20 (1H, s), 7.44 (1H, s), 8.25-8.28 (1H, d, J = 8.8 Hz). ESI-TOF-MS: *m/z* for $C_{33}H_{43}N_2O_8$: calcd 595.30 $[M+H]^+$, observed 595.31.

Synthesis of 9

To a cooled (0 °C) solution of **8** (780 mg, 1.32 mmol) in THF-MeOH (1 : 1, 20 mL) was added dropwise 1*N* NaOH (10 mL). The reaction mixture was stirred at 0 °C for 60 min and then neutralized with 1*N* HCl. After dilution with $CHCl_3$. The organic layers were washed with brine. The solvent was removed in vacuo to give **9** (626 mg) as a yellow solid. This material was used for the next step without further purification.

^{1}H-NMR (400 MHz, CD_3OD) δ 1.49 (9H, s), 3.30 (4H, br), 3.63 (2H, br), 4.22 (2H, br), 4.74 (2H, s), 6.84-6.85 (1H, d, J = 2.0 Hz), 6.90-6.92 (1H, dd, J = 2.4 Hz, 8.8 Hz), 6.98 (1H, d, J = 2.4 Hz), 7.01-7.02 (1H, d, J =2.8 Hz), 8.12-8.14 (1H, d, J =9.2 Hz). ESI-TOF-MS: *m/z* for $C_{23}H_{27}N_2O_6$: calcd 427.19 $[M+H]^+$, observed 427.19.

Synthesis of 10

To a cooled (0 °C) solution of **9** (626 mg, 1.3 mmol) and pyridine (1.06 mL, 13.2 mmol) in dry CH_2Cl_2 (5 mL) was added dropwise a solution of trifluoromethanesulfonic anhydride (Tf_2O, 1.10 mL, 6.6 mmol) in dry CH_2Cl_2 (20 mL). The reaction mixture was stirred at rt for 60 min. After quenching the reaction with saturated $NaHCO_3$, the mixture was extracted with $CHCl_3$. The organic layers were washed with saturated $NaHCO_3$ and dried over Na_2SO_4. After removal of the solvent in vacuo, the residue was purified by flash column chromatography on silica gel ($CHCl_3$ / MeOH = 50 / 1) to give **10** (780 mg, 86%) as a brown solid.

^{1}H-NMR (400 MHz, $CDCl_3$). δ 1.48 (9H, s), 2.55 (4H, s), 3.50 (4H, s), 4.31 (2H, s), 7.31-7.33 (1H, dd, J = 2.4 Hz, 8.8 Hz), 7.35-7.36 (1H, d, J = 2.4 Hz), 7.44-7.45 (1H, d, J = 2.4 Hz), 7.84 (1H, s), 8.36-8.38(1H, d, J = 9.2 Hz). ESI-TOF-MS: *m/z* for $C_{25}H_{25}F_6N_2O_{10}S_2$: calcd 691.08 $[M+H]^+$, observed 691.08.

Synthesis of 16

A solution of **10** (200 mg, 0.29 mmol) and pyrrolidine (24 μL, 0.29 mmol) in dry DMSO (5 mL) was stirred at 85 °C for 2 h. After cooling to rt, the reaction mixture was diluted with $CHCl_3$ and washed with saturated $NaHCO_3$ followed by drying over Na_2SO_4. After removal of the solvent in vacuo, the residue

was purified by flash column chromatography on silica gel ($CHCl_3$ / MeOH = 50 / 1) to give **16** (104.4 mg) as a yellow oil. This material was used for the next step without separation of the isomers. A single isomer **16'** was purified by flash column chromatography on silica gel (hexane / AcOEt = 5 /1 → 2 / 1) for spectroscopic measurements.

^{1}H-NMR (400 MHz, $CDCl_3$). δ 1.48 (9H, s), 2.09 (4H, brs), 2.55(4H, brs), 3.43 (4H, brs), 3.50 (4H, brs), 4.39 (2H, s), 6.32 (1H, s), 6.60-6.63 (1H, d, J = 9.2 Hz), 7.23 (1H, s), 7.73 (1H, s), 8.04-8.06 (1H, d, J = 8.8 Hz). ESI-TOF-MS: m/z for $C_{28}H_{33}F_3N_3O_7S$: calcd 612.20 $[M+H]^+$, observed 612.20.

Synthesis of 11

A solution of **16** (mixture of the isomers, 103 mg, 0.17 mmol) and piperidine (84.0 μL, 0.85 mmol) in dry DMSO (5 mL) was stirred at 85 °C for 18 h. After cooling to rt, the reaction mixture was diluted with $CHCl_3$ and washed with saturated $NaHCO_3$ followed by drying over Na_2SO_4. After removal of the solvent in vacuo, the residue was purified by flash column chromatography on silica gel ($CHCl_3$ / MeOH = 50 / 1) to give a mixture of **11** (63.3 mg, 40%, 2step) as a yellow oil. A single isomer **11'** was synthesized from **16'** by the same reaction for the spectroscopic measurements.

^{1}H-NMR (400 MHz, $CDCl_3$). δ 1.47 (9H, s), 1.69 (6H, brs), 2.06-2.09 (4H, m), 2.57-2.61 (4H, m), 3.40-3.41 (8H, m), 3.47-3.49 (4H, m), 4.36 (2H, s), 6.29 (1H, s), 6.53-6.55 (1H, d, J = 9.2 Hz), 6.60 (1H, s), 7.25 (1H, s), 8.03-8.06 (1H, d, J = 9.2 Hz); ESI-TOF-MS: m/z for $C_{32}H_{43}N_4O_4$: calcd 547.33 $[M+H]^+$, observed 547.32.

Synthesis of 12

To a solution of **11** (mixture of the isomers, 63.3 mg, 116.0 μmol) in dry THF (5 mL) was added borane dimethylsulfide complex (1.27 mL, 1160 μmol). The reaction mixture was stirred at 50 °C for 2 h. After cooling to rt, the reaction was quenched with water. The mixture was diluted with $CHCl_3$ and washed with saturated $NaHCO_3$ and brine followed by drying over Na_2SO_4. After removal of the solvent in vacuo, the residue was purified by flash column chromatography on silica gel ($CHCl_3$) to give **12** (43.8 mg, 71%) as a yellow oil. A single isomer **12'** was synthesized from **11'** by the same reaction for spectroscopic measurements.

^{1}H-NMR (400 MHz, $CDCl_3$). δ 1.41 (9H, s), 1.57-1.62 (6H, m), 1.69-1.73 (4H, m), 1.99-2.02 (4H, m), 3.15-3.17 (4H, t, J = 5.2 Hz), 3.26-3.29 (4H, m), 3.38-3.43 (4H, m), 3.87 (2H, s), 4.17 (2H, brs), 6.24 (1H, d, J = 2.4 Hz), 6.29-6.31 (1H, dd, J = 2.4 Hz, 8.4 Hz), 6.56-6.57 (1H, d, J = 2.8 Hz), 6.68 (1H, d, J = 2.4 Hz), 7.00-7.02 (1H, d, J = 8.4 Hz). ESI-TOF-MS: m/z for $C_{32}H_{45}N_4O_3$: calcd 533.35 $[M+H]^+$, observed 533.35.

Synthesis of 13

To a solution of **12** (43.8 mg, 82.0 mmol) in dry EtOH - CH_2Cl_2 (1 : 2, 9 mL) was added a solution of DDQ (38.6 mg, 411.0 μmol) in dry EtOH (3 mL). The reaction mixture was stirred at rt for 1 h. The mixture was diluted with $CHCl_3$ and washed with saturated $NaHCO_3$ and brine followed by drying over Na_2SO_4. After removal of solvent in vacuo, the residue was purified by flash column chromatography on silica gel ($CHCl_3$ / MeOH / NH_3 = 200 / 10 / 1) to give **13** (15.4 mg, 35%) as a red powder. A single isomer **13'** was synthesized from **12'** by the same reaction for spectroscopic measurements.

^{1}H-NMR (400 MHz, CD_3OD). δ 1.45 (9H, s), 1.60-164 (2H, m), 1.69-1.74 (4H, m), 2.01-2.05 (4H, m), 2.42-2.48 (4H, m), 3.20-3.22 (4H, m), 3.29-3.31 (4H, m), 3.40-3.45 (4H, m), 3.63 (2H, s), 6.04 (1H, s), 6.28 (1H, d, J = 2.4 Hz), 6.42-6.45 (1H, dd, J = 2.4 Hz, 8.8 Hz), 6.59 (1H, d, J = 2.4 Hz), 6.84 (1H, d, J = 2.4 Hz), 7.24-7.26 (1H, d, J = 8.4 Hz). ESI-TOF-MS: *m/z* for $C_{32}H_{43}N_4O_3$: calcd 531.33 $[M]^+$, observed 531.33.

Synthesis of 15

To a solution of **13** (4.9 mg, 9.2 μmol) in CH_2Cl_2 (2 mL) was added trifluoroacetic acid (2 mL). The reaction mixture was stirred at rt for 2 h and then concentrated in vacuo. After co-evaporated with $CHCl_3$ (×2), the residue dissolved in dry DMF (3 mL) was mixed with DIEA (32 μL, 184.0 μmol) and **14** (9.8 mg, 18.4 μmol). The mixture was stirred at rt for 38 h. The mixture was diluted with $CHCl_3$, washed with water and brine, followd by drying over Na_2SO_4. After removal of the solvent in vacuo, the residue was purified by flash column chromatography on silica gel ($CHCl_3$ / MeOH / NH_3 = 200 / 10 / 1) to give **15** (2.6 mg, 34%) as a red solid. A single isomer **15'** was synthesized from **13'** by the same reaction for spectroscopic measurements.

^{1}H-NMR (400MHz, CD_3OD) δ 1.48 (18H, s), 1.76 (6H, brs), 2.16 (4H, brs), 2.58 (2H, brs), 2.64 (2H, brs), 3.45 (4H, brs), 3.64 (4H, brs), 3.90 (2H, s), 4.20 (4H, s), 6.48 (1H, d, J = 2.0 Hz), 6.65-6.67 (1H, d, J = 8.8 Hz), 6.76 (1H, s), 7.06-7.08 (1H, d, J = 8.0 Hz), 7.29 (1H, s), 7.50-7.52 (1H, d, J = 8.8 Hz), 7.84-7.86 (1H, d, J = 9.2 Hz), 7.94 (1H, s), 9.04 (1H, s). ESI-TOF-MS: *m/z* for $C_{49}H_{60}N_5O_8$: calcd 846.44 $[M]^+$, observed 846.44.

Synthesis of 6

To a solution of **15** (2.6 mg, 3.1 μmol) in CH_2Cl_2 (2 mL) was added trifluoroacetic acid (2 mL). The reaction mixture was stirred at rt for 8 h. After removal of the solvent in vacuo, the residue was purified by reverse-phase HPLC to give **6** (1.9 mg, 72% as monoTFA salt. A single isomer **6'** was synthesized from **15'** by the same reaction for spectroscopic measurements.

HPLC conditions

Column: YMC-Triart C18 10 mm × 250 mm, Flow rate: 3.0 mL/min, Detection: UV(220 nm)

A : MeCN (0.1% TFA), B : H_2O (0.1% TFA)

A / B = 20 / 80 (0 min) → 50 / 50 (40 min) → 100 / 0 (50 min) → 20 / 80 (60 min)

^{1}H-NMR (400MHz, CD_3OD) δ1.78 (6H, brs), 2.16 (4H, brs), 3.08 (4H, brs), 3.65 (8H, brs), 3.78 (4H, brs), 4.31 (4H, s), 4.36 (2H, s), 6.54 (1H, s), 6.71-6.73 (1H, d, J = 8.8 Hz), 6.79 (1H, s), 7.10 (1H, s), 7.10-7.12 (1H, d, J = 9.2 Hz), 7.45 (1H, s), 7.52-7.54 (1H, d, J = 8.8 Hz), 7.83-7.85 (1H, d, J = 9.2 Hz), 8.01 (1H, s), 8.99 (1H, s). ESI-TOF-MS: *m/z* for $C_{41}H_{44}N_5O_8$: calcd 734.3184 $[M]^+$, observed 734.3184.

Scheme S2. Synthesis of 6-AM

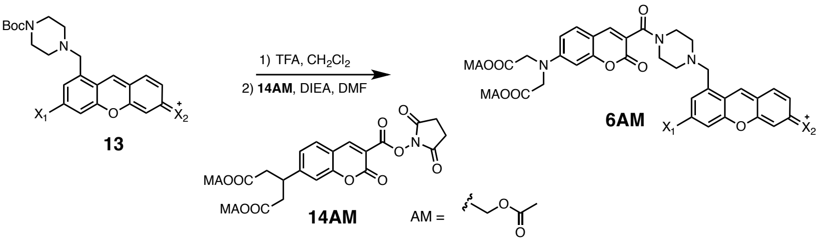

Synthesis of 6-AM

To a solution of **13** (15.4 mg, 29.0 μmol) in CH_2Cl_2 (2 mL) were added trifluoroacetic acid (2 mL). The reaction mixture was stirred at rt for 2 h and then the solvent was evaporated in vacuo. After co-evaporated with $CHCl_3$ (×2), the residue dissolved in dry DMF (3 mL) was mixed with DIEA (48.7 μL, 290.0 μmol) and **14**-AM (13.0 mg, 23.2 μmol) nad stirredf at rt for 20 h. An excess **14-AM** (24.9 mg 44.4 μmol) was added and the mixture was further stirred at rt for 48 h. After dilution with $CHCl_3$, the organic layers were washed with water and brine followed by drying over Na_2SO_4. The solvent was removed in vacuo, and the residue was purified by reverse-phase HPLC to give **6**-AM (8.9 mg, 35%) as a red solid.

HPLC conditions

Column: YMC-Triart C18 10 mm × 250 mm, Flow rate: 3.0 mL/min, Detection: UV(220 nm)

A : MeCN (0.1% TFA), B : H_2O (0.1% TFA)

A / B = 5 / 95 (0 min) → 35 / 65 (10 min) → 55 / 45 (50 min) → 100 / 0 (60 min) → 5 / 95 (70 min)

^{1}H-NMR (400MHz, CD_3OD) δ 2.07-2.08 (2H, m), 2.08 (6H, s), 2.15-2.17 (4H, m), 3.12-3.14 (4H, m), 3.65 (8H, br), 3.80-3.82 (4H, m), 4.41 (4H, s), 4.43 (2H, s), 5.80 (4H, s), 6.57 (1H, s), 6.73-6.75 (1H, d, J = 8.8 Hz), 6.81 (1H, s), 7.10 (1H, s), 7.21 (1H, s), 7.34-7.37 (1H, d, J = 12.0 Hz), 7.53-7.55 (1H, d, J = 8.8 Hz), 7.82-7.84 (1H, d, J = 9.2 Hz), 8.03 (1H, s), 8.99 (1H, s); ESI-TOF-MS *m/z* found 878.34 for $C_{47}H_{52}N_5O_{12}$ $[M+H]^+$. ESI-TOF-MS: *m/z* for $C_{47}H_{52}N_5O_{12}$: calcd 878.3607 $[M]^+$, observed 878.3607.

Scheme S3. Synthesis of 1

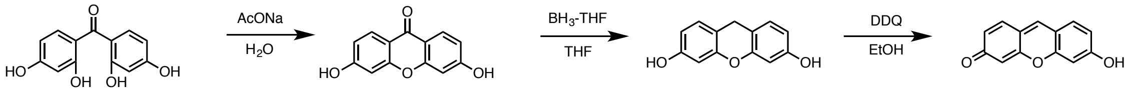

Synthesis of 18

A mixture of **17** (9.97 g, 40.5 mmol) and AcONa (33.3 g, 406.0 mmol) in water (250 mL) was refluxed for 24 h. After cooled to 0 °C, the solution was neutralized with conc. HCl. The precipitation was filterated and washed with water. The filtered residue was dried in vacuo at 50 °C for 2 h to give **18** as a white solid (9.22 g, 99%).

^{1}H-NMR (400MHz, DMSO-d6) δ 6.82 (2H, d, J = 2.0 Hz), 6.85-6.88 (2H, d, J = 8.8 Hz), 7.97-7.99 (2H, d, J = 8.8 Hz), 10.79 (2H, br). ESI-TOF-MS: *m/z* for $C_{13}H_7O_4$: calcd 227.04 $[M-H]^-$, observed 227.04.

Synthesis of 19

18 (100 mg, 438 μmol) was added into a solution of BH_3-THF (2.7 mmol) in dry THF (10 mL). The solution was stirred at rt for 5 h. TMSCl (80 μL, 633 μmol) was added and the solution was further stirred at rt for 1.5 h. The reaction was quenched with water. After dilution with AcOEt, the organic layer was washed with water and brine followed by drying over Na_2SO_4. After removal of the solvent in vacuo, the residue was purified by flash column chromatography on silica gel ($CHCl_3$ / MeOH / NH_3 = 200 / 10 / 1) to give **19** (59.5 mg 63%) as an orange solid.

^{1}H-NMR (400MHz, DMSO-d6) δ 3.78 (2H, s), 6.41 (2H, d, J = 2.0 Hz), 6.47-6.49 (2H, d, J = 8.4 Hz), 6.98-7.00 (2H, d, J = 8.4 Hz), 9.41 (2H, s). ESI-TOF-MS: *m/z* for $C_{13}H_9O_3$: calcd 213.06 $[M-H]^-$, observed 213.05.

Synthesis of 1

To a solution of **19** (56.5 mg, 264 μmol) in dry CH_2Cl_2-EtOH (1 : 2 , 6 mL) was added a solution of DDQ (66.3 mg, 290 μmol) in dry EtOH (4.5 mL). The solution was stirred at rt for 2 h. The solvent was removed in vacuo and the residue was purified in flash column chromato graphy on silica gel ($CHCl_3$ / MeOH / NH_3 = 80 / 10 / 1) to give **1** (50.3 mg, 90%) as a purple solid.

^{1}H-NMR (400MHz, DMSO-d6) δ 6.48 (2H, br), 6.67 (2H, br), 7.59-7.61 (2H, d, J =9.2 Hz), 8.20 (1H, s), 11.06 (1H, br). ESI-TOF-MS: *m/z* for $C_{13}H_7O_3$: calcd 211.0401 [M-H]$^-$, observed 211.0403.

Scheme S4. Synthesis of 2

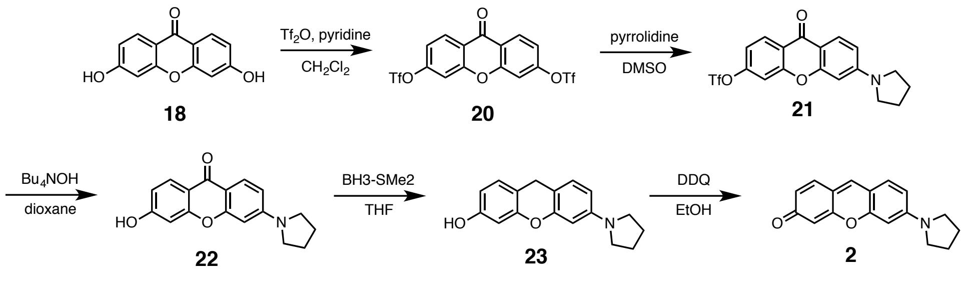

Synthesis of 20

To a cooled (0 °C) solution of **18** (300 mg, 1.3 mmol) and pyridine (1.07 mL, 13.1 mmol) in dry CH_2Cl_2 (5 mL) was added dropwise a solution of trifluoromethanesulfonic anhydride (Tf_2O, 1.12 mL, 6.6 mmol) in dry CH_2Cl_2 (15 mL). The reaction mixture was stirred at rt for 60 min. After quenching with saturated $NaHCO_3$, the mixture was extracted with $CHCl_3$. The organic layers were washed with saturated $NaHCO_3$ and dried over Na_2SO_4. After removal of the solvent in vacuo, the residue was purified by flash column chromatography on silica gel ($CHCl_3$ / MeOH = 50 / 1) to give **20** (318 mg, 49%) as a white solid.

^{1}H-NMR (400 MHz, $CDCl_3$). δ 7.34-7.37 (2H, dd, J = 2.4 Hz, 8.8 Hz), 7.50 (2H, d, J = 2.0 Hz), 8.44-8.47 (2H, d, J = 8.8 Hz). ESI-TOF-MS: *m/z* for $C_{15}H_6F_6NaO_8S_2$: calcd 514.93 [M+Na]$^+$, observed 514.93.

Synthesis of 21

A solution of **20** (200 mg, 410 μmol) and pyrrolidine (27.3 μL, 330 μmol) in dry DMSO (3 mL) was stirred at 85 °C for 14 h. After dilution with $CHCl_3$, the mixture was washed with saturated $NaHCO_3$ and brine followed by drying over Na_2SO_4. The solvent was removed in vacuo and the residue was purified by flash column chromatography on silica gel ($CHCl_3$) to give **21** (80.7 mg, 59%) as an orange solid.

^{1}H-NMR (400MHz, $CDCl_3$) δ 2.08-2.11 (4H, m), 3.42-3.46 (4H, m), 6.38 (1H, d, J = 2.0 Hz), 6.62-6.65 (1H, dd, J = 2.0 Hz, 8.8 Hz), 7.20-7.23 (1H, dd, J = 2.0 Hz, 8.8 Hz), 7.35 (1H, d, J = 2.8 Hz), 8.13-8.15 (1H, d, J = 9.2 Hz), 8.38-8.40 (1H, d, J = 8.4 Hz). ESI-TOF-MS: *m/z* for $C_{18}H_{14}F_3NNaO_5S$: calcd: 436.04 $[M+Na]^+$, observed 436.04.

Synthesis of 22

To a solution of **21** (80.7 mg, 200 μmol) in dry 1,4-dioxane (5 mL) was added dropwised a 10 % MeOH solution of $^tBu_4NOH$ (1 mL, 400 μmol). The reaction mixture was stirred at rt for 1 h. The mixture was diluted with $CHCl_3$ and washed with saturated $NaHCO_3$ and brine followed by drying over Na_2SO_4. After removal of the solvent in vacuo, the residue was purified by flash column chromatography on silica gel ($CHCl_3$ / MeOH = 30 / 1) to give **22** (37.7 mg, 67%) as a red solid.

^{1}H-NMR (400MHz, DMSO-d6) δ 2.00 (4H, s), 3.38 (4H, s), 6.44 (1H, s), 6.65-6.67 (1H, d, J = 8.4 Hz), 6.77 (1H, s), 6.80-6.82 (1H, d, J = 8.8 Hz), 7.89-7.91 (1H, d, J = 9.2 Hz), 7.93-7.95 (1H, d, J = 8.8 Hz). ESI-TOF-MS: *m/z* for $C_{17}H_{15}NNaO_3$: calcd: 304.09 $[M+Na]^+$, observed 304.09.

Synthesis of 23

To a solution of borane-THF complex (0.40 mL, 356 μmol) in dry THF (2 mL) was added dropwised a solution of **22** (20.0 mg, 71.1 μmol) in dry THF (5 mL). The reaction mixture was stirred at 50 °C for 16 h. After cooling to rt, the reaction was quenched with water. The mixture was diluted with AcOEt and washed with saturated $NaHCO_3$ and brine followed by drying over Na_2SO_4. After removal of the solvent in vacuo, the residue was purified by flash column chromatography on silica gel ($CHCl_3$) to give **23** (15.0 mg, 79%) as a pink solid.

^{1}H-NMR (400MHz, CD_3OD) δ 2.01 (4H, brs), 3.25 (4H, brs), 3.80 (2H, s), 6.20 (1H, s), 6.30-6.32 (1H, d, J = 7.6 Hz), 6.43 (1H, s), 6.45-6.47 (1H, d, J = 8.4 Hz). 6.95-6.97 (1H, d, J = 8.4 Hz). ESI-TOF-MS: *m/z* for $C_{17}H_{16}NO_2$: calcd: 266.12 $[M-H]^-$, observed 266.12.

Synthesis of 2

To a solution of **23** (15.0 mg, 56.2 μmol) in dry EtOH-CH_2Cl_2(1 : 2, 9 mL) was added a solution of DDQ (15.9 mg, 168.6 μmol) in dry EtOH (3 mL). The reaction mixture was stirred at rt for 2 h. After removal of solvent in vacuo, the residue was purified by flash column chromatography on silica gel ($CHCl_3$ / MeOH = 30 / 1 → 15 / 1) to give **2** (8.7 mg, 58%) as a red solid.

^{1}H-NMR (400MHz, CD_3OD) δ 2.12 (4H, brs), 3.54 (4H, brs), 6.44 (1H, s), 6.67 (1H, s), 6.67-6.69 (1H, d, J = 8.8 Hz), 6.86-6.88 (1H, d, J = 8.8 Hz). 7.56-7.59 (1H, d, J = 9.2 Hz), 7.64-7.66 (1H, d, J = 8.8 Hz). 8.28 (1H, s). ESI-TOF-HRMS: *m/z* for $C_{17}H_{16}NO_2$: calcd: 266.1176 $[M+H]^+$; observed 266.1167.

Scheme S5. Synthesis of 3

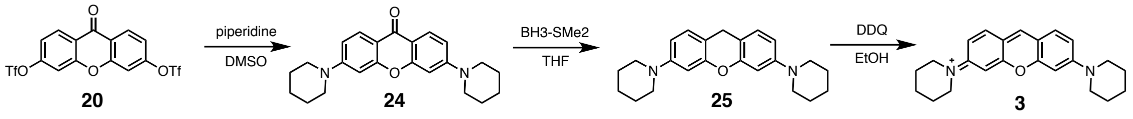

Synthesis of 24

A solution of **20** (150 mg, 310 μmol) and piperidine (260 μL, 3100 μmol) in dry DMSO (5 mL) was stirred at 85°C for 20 h. After dilution with $CHCl_3$, the mixture was washed with saturated $NaHCO_3$ and brine followed by drying over Na_2SO_4. The solvent was removed in vacuo and the residue was purified by flash column chromatography on silica gel ($CHCl_3$) to give **24** (100 mg, 90%) as an orange solid.

^{1}H-NMR (400MHz, $CDCl_3$) δ 1.68-1.70 (12H, m), 3.39-3.41 (8H, m), 6.67 (2H, d, J = 2.4 Hz), 6.86-6.88 (2H, d, J = 2.4 Hz, 9.2 Hz), 8.10-8.12 (2H, d, J = 8.8 Hz). ESI-TOF-MS: *m/z* for $C_{23}H_{26}N_2NaO_2$: calcd 385.19 $[M+Na]^+$, observed 385.19.

Synthesis of 25

A Solution of **24** (50 mg, 138 μmol) in dry THF (5 mL) was added a solution of borane-SMe_2 complex (770 μL, 690 μmol). The reaction mixture was stirred at 50 °C for 1 h. After cooling to rt, the reaction was quenched with water. The mixture was diluted with $CHCl_3$ and washed with saturated $NaHCO_3$ and brine followed by drying over Na_2SO_4. After removal of the solvent in vacuo, the residue was purified by flash column chromatography on silica gel ($CHCl_3$) to give **25** (42mg, 86%) as a red solid.

^{1}H-NMR (400MHz, $CDCl_3$) δ 1.69-1.71 (12H, m), 3.13-3.15 (8H, m), 6.58 (2H, s), 6.62-6.64 (2H, d, J = 8.4 Hz), 6.99-7.01 (2H, d, J = 8.8 Hz). ESI-TOF-MS: *m/z* for $C_{23}H_{29}N_2O$: calcd 349.23 $[M+H]^+$, observed 349.22.

Synthesis of 3

To a solution of **25** (42 mg, 119 μmol) in dry EtOH-CH_2Cl_2(1 : 2.5, 7 mL) was added a solution of DDQ (32 mg,360 μmol) in dry EtOH (3 mL). The reaction mixture was stirred at rt for 40 min. The mixture was diluted with $CHCl_3$ and washed with saturated $NaHCO_3$ and brine followed by drying over

Na_2SO_4. After removal of solvent in vacuo, the residue was purified by flash column chromatography on silica gel ($CHCl_3$ / MeOH / NH_3 = 100 / 10 / 1) to give **3** (16 mg, 39%) as a red solid. ^{1}H-NMR was observed as a MeOH adduct.

^{1}H-NMR (400MHz, CD_3OD) δ 1.70-1.70 (12H, m), 3.21-3.24 (8H, m), 5.53 (1H, s), 6.68 (2H, d, J = 2.4 Hz), 6.79-6.83 (2H, dd, J = 2.8 Hz, 8.4 Hz), 7.30-7.33 (2H, d, J = 8.4 Hz). ESI-TOF-HRMS: *m/z* for $C_{23}H_{27}N_2O$: calcd 347.2118 $[M]^+$, observed 347.2135.

Scheme S6. Synthesis of 4

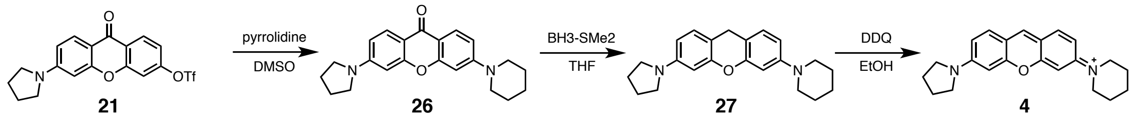

Synthesis of 26

A solution of **21** (70 mg, 169 μmol) and pyrrolidine (85.4 μL, 847 μmol) in dry DMSO (3 mL) was stirred at 85°C for 14 h. After dilution with $CHCl_3$, the mixture was washed with saturated $NaHCO_3$ and brine followed by drying over Na_2SO_4. The solvent was removed in vacuo and the residue was purified by flash column chromatography on silica gel ($CHCl_3$ / MeOH = 50 / 1) to give **26** (80.7 mg, 59%) as an orange solid.

^{1}H-NMR (400MHz, $CDCl_3$) δ 1.69 (6H, s), 2.05-2.07 (4H, m), 3.41 (8H, s), 6.34 (1H, s), 6.55-6.57 (1H, d, J = 8.8 Hz), 6.68 (1H, s), 6.86-6.88 (1H, d, J = 8.8 Hz), 8.11-8.13 (2H, d, J = 9.2 Hz). ESI-TOF-MS: *m/z* for $C_{22}H_{24}N_2NaO_2$: calcd 371.17 $[M+Na]^+$, observed 371.16.

Synthesis of 27

To a solution of **26** (30.0 mg, 86,2 μmol) in dry THF (3 mL) was added borane-THF complex (0.48 mL, 430 μmol). The reaction mixture was stirred at 50 °C for 2 h. After cooling to rt, the reaction was quenched with water. The mixture was diluted with AcOEt and washed with saturated $NaHCO_3$ and brine followed by drying over Na_2SO_4. After removal of the solvent in vacuo, the residue was purified by flash column chromatography on silica gel ($CHCl_3$) to give **27** (27.3 mg, 95%) as a red solid.

^{1}H-NMR (400MHz, $CDCl_3$) δ 1.57-1.59 (2H, m), 1.69-1.71 (4H, m), 1.98-2.02 (4H, m), 3.13-3.16 (4H, m), 3.26-3.29 (4H, m), 3.86 (2H, s), 6.25 (1H, s), 6.27-6.29 (1H, d, J = 8.0 Hz), 6.60 (1H, s), 6.62-6.64 (1H, d, J = 8.4 Hz), 6.97-7.02 (2H, m). ESI-TOF-MS: *m/z* for $C_{22}H_{27}N_2O$: calcd 335.21 $[M+H]^+$, observed 335.21.

Synthesis of 4

To a solution of **27** (27.3 mg, 81.7 μmol) in dry EtOH-CH_2Cl_2 (1 : 2, 9 mL) was added a solution of DDQ (15.9 mg, 168.6 μmol) in dry EtOH (3 mL). The reaction mixture was stirred at rt for 1 h. After removal of solvent in vacuo, the residue was purified by flash column chromatography on silica gel ($CHCl_3$ / MeOH = 30 / 1 → 15 / 1) to give **4** (6.7 mg, 25%) as a red solid.

^{1}H-NMR (400MHz, CD_3OD) δ1.76 (6H, brs), 2.15 (4H, s), 3.44 (4H, brs), 3.78 (4H, s), 6.76 (1H, s), 7.03-7.06 (1H, d, J = 11.2 Hz), 7.06 (1H, s), 7.28-7.31 (1H, d, J = 9.6 Hz), 7.76-7.78 (2H, d, J = 9.6 Hz), 8.52 (1H, s). ESI-TOF-HRMS: *m/z* for $C_{22}H_{25}N_2O$: calcd 333.1961 $[M]^+$, observed 333.1966.

Scheme S7. Synthesis of 5

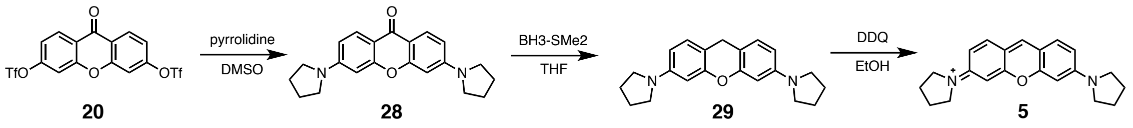

Synthesis of 28

A solution of **20** (300 mg, 610 μmol) and pyrrolidine (255 μL, 3050 μmol) in dry DMSO (5 mL) was stirred at 85°C for 17 h. After dilution with $CHCl_3$, the mixture was washed with saturated $NaHCO_3$ and brine followed by drying over Na_2SO_4. The solvent was removed in vacuo and the residue was purified by flash column chromatography on silica gel ($CHCl_3$) to give **28** (132 mg, 56%) as an orange solid.

^{1}H-NMR (400MHz, $CDCl_3$) δ 2.06-2.08 (8H, m), 3.39-3.41 (8H, m), 6.35 (2H, d, J = 2.4 Hz), 6.55-6.57 (2H, dd, J = 2.4 Hz, 8.8 Hz), 8.11-8.14 (2H, d, J = 8.8 Hz). ESI-TOF-MS: *m/z* for $C_{21}H_{23}N_2O_2$: calcd 335.18 $[M+H]^+$, observed 335.18.

Synthesis of 29

To a solution of Borane dimethylsulfide complex (0.40 mL, 356 μmol) in dry THF (2 mL) was added dropwised a solution of **28** (132 mg, 395 μmol) in dry THF (10 mL). The reaction mixture was stirred at 45 °C for 1 h. After cooling to rt, the reaction was quenched with water. The mixture was diluted with AcOEt and washed with saturated $NaHCO_3$ and brine followed by drying over Na_2SO_4. After removal of the solvent in vacuo, the residue was purified by flash column chromatography on silica gel ($CHCl_3$) to give **29** (120 mg, 95%) as a red solid.

^{1}H-NMR (400MHz, $CDCl_3$) δ 1.98-2.01 (8H, m), 3.26-3.29 (8H, m), 3.86 (2H, s), 6.26-6.29 (4H, m), 6.98-7.00 (2H, d, J = 8.0 Hz). ESI-TOF-MS: *m/z* for $C_{21}H_{25}N_2O$: calcd 321.20 $[M+H]^+$, observed 321.19.

Synthesis of 5

To a solution of **29** (24.7 mg, 77.1 μmol) in dry EtOH-CH_2Cl_2(1 : 2, 9 mL) was added a solution of DDQ (20.1 mg, 213.6 μmol) in dry EtOH (3 mL). The reaction mixture was stirred at rt for 2 h. After removal of solvent in vacuo, the residue was purified by flash column chromatography on silica gel ($CHCl_3$ / MeOH = 20 / 1 → 10 / 1) to give **5** (10.7 mg, 47%) as a red solid.

^{1}H-NMR (400MHz, CD_3OD) δ 2.14-2.17 (8H, m), 3.65 (8H, brs), 6.80 (2H, s), 7.04-7.07 (1H, dd, J = 2.4 Hz, 9.2 Hz), 7.78-7.80 (2H, d, J = 9.2 Hz), 8.57 (1H, s). ESI-TOF-HRMS: m/z for $C_{21}H_{23}N_2O$: calcd 319.1802 $[M]^+$, observed 319.1817.

Scheme S8. Synthesis of 7

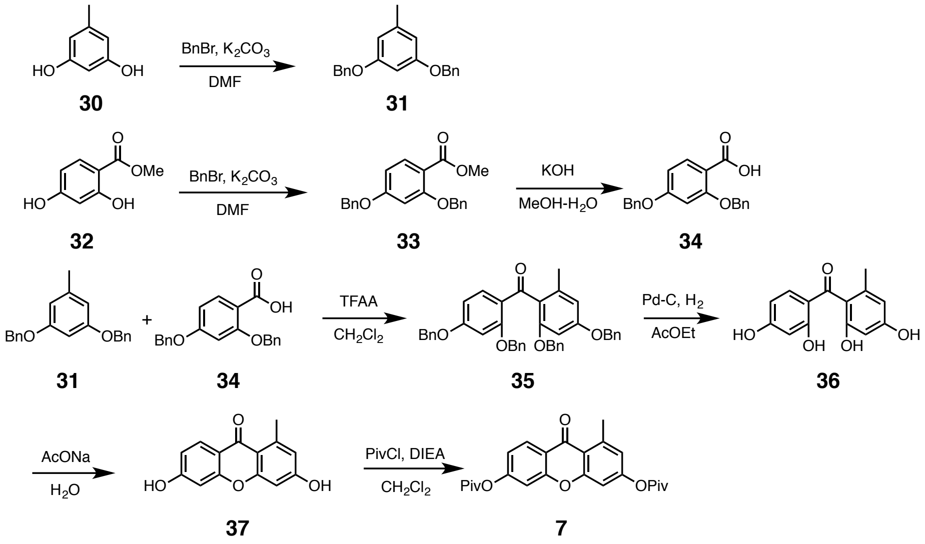

Synthesis of 31

To a solution of 5-methylresorcinol **32** (10.3 g, 80.6 mmol,) and K_2CO_3 (33.1 g, 239.5 mmol) in dry DMF (50 mL) was added benzyl bromide (19.2 mL, 160.5 mmol). The mixture was stirred at 70 °C for 4 h. After filtration, the filtrate was diluted with AcOEt and washed with saturated $NaHCO_3$, water and brine followed by drying over Na_2SO_4. After removal of the solvent in vacuo, the residue was purified by flash column chromatography on silica gel (hexane / AcOEt = 40 / 1) to give **31** (21.2 g, 86%) as a colorless liquid.

^{1}H-NMR (400MHz, $CDCl_3$) δ 2.30 (3H, s), 5.04 (4H, s), 6.47 (3H, s), 7.31-7.42 (10H, m). ESI-TOF-MS: m/z for $C_{21}H_{20}NaO_2$: calcd 327.14 $[M+Na]^+$, observed 327.13.

Synthesis of 33

To a solution of methyl-2,4-dihydroxybenzoate **32** (5.0 g, 29.8 mmol) and K_2CO_3 (14.3 g, 103.5 mmol,) in dry DMF (50 mL) was added benzyl bromide (10.0 mL, 83.6 mmol). The mixture was stirred at 70°C for 10 h. After filtration, the filtrate was diluted with AcOEt and washed with saturated $NaHCO_3$, water and brine followed by drying over Na_2SO_4. After removal of the solvent in vacuo, the residue was washed with diisopropyl ether by filttration to give **33** (8.8 g, 85%) as a white solid.

^{1}H-NMR (400MHz, $CDCl_3$) δ 3.88 (3H, s), 5.07 (2H, s), 5.14 (2H, s), 6.58-6.62 (2H, m), 7.29-7.41 (8H, m), 7.49-7.51 (2H, d, J = 7.6 Hz), 7.87-7.89 (1H, d, J = 8.4 Hz). ESI-TOF-MS: *m/z* for $C_{22}H_{20}NaO_4$: calcd 371.13 $[M+Na]^+$, observed 371.13.

Synthesis of 34

A solution of **33** (10.9 g, 31.3 mmol) and KOH (36.2 g, 646.2 mmol) in EtOH-H2O (1 : 1 200 mL) was refluxed for 13 h. After cooling to rt, the solution was acidified to pH 4 with conc. HCl. The mixture was diluted with $CHCl_3$ and the organic layers were washed with water and brine followed by drying over Na_2SO_4. The solvent was removed in vacuo to give **34** (10.7 g, 97%) as a white solid.

^{1}H-NMR(400MHz, $CDCl_3$) δ 5.11 (2H, s), 5.23 (2H, s), 6.69 (1H, s), 6.72-6.74 (1H, d, J = 8.8 Hz), 7.38-7.42 (10H, m), 8.14-8.16 (1H, d, J = 9.2 Hz). ESI-TOF-MS: *m/z* for $C_{21}H_{18}NaO_4$: calcd 357.11 $[M+Na]^+$, observed 357.11.

Synthesis of 35

To a cooled (0 °C) solution of **34** (10.2 g, 30.5 mmol,) and **31** (34.3 g, 112.9 mmol) in dry CH_2Cl_2 (150 mL) was added dropwise TFAA (15.0 mL). The mixture was stirred at rt for 1.5 h. After removal of the solvent, the residue was purified by flash column chromatography on silica gel (toluene / hexane =1 / 1 → $CHCl_3$ / hexane = 1 / 1 → $CHCl_3$) to give **35** (17.1 g, 90%) as a white solid.

^{1}H-NMR (400MHz, $CDCl_3$) δ 2.20 (3H, s), 4.82 (4H, s), 4.96 (2H, s), 5.08 (2H, s), 6.30-6.31 (2H, d, J = 6.4 Hz), 6.53 (1H, d, J = 2.0 Hz), 6.58-6.61 (1H, dd, J = 2.4 Hz, 8.4 Hz), 6.95-6.97 (2H, d, J = 8.4 Hz), 7.03-7.04 (2H, m), 7.12-7.20 (4H, m), 7.22-7.24 (2H, m), 7.35-7.44 (10H, m), 7.73-7.75 (1H, d, J = 8.8 Hz). ESI-TOF-MS: *m/z* for $C_{42}H_{36}NaO_5$: calcd 643.25 $[M+Na]^+$, observed 643.25.

Synthesis of 36

To a solution of compound **35** (17,1 g, 27.6 mmol) in AcOEt (250 mL) was added 10% Pd/C (2.0 g) under N_2 atmosphere. The nitorgen gas was degassed and backfilled with H_2 gas twice. The solution was

stirred at rt for 24 h. After removal of Pd/C by filtration, the solvent was washed with AcOEt to give **36** (7.0 g, 97%) as a yellow amorphous powder.

^{1}H-NMR(400MHz, DMSO-d6) δ 1.96(3H, s), 6.15(1H, s), 6.20(1H, s), 6.26-6.29 (2H, m), 7.08-7.10 (1H, d, J = 8.8 Hz), 9.48 (2H, br), 10.62 (1H, br), 12.73 (1H, s). ESI-TOF-MS: m/z for $C_{14}H_{12}NaO_5$: calcd 283.06 $[M+Na]^+$, observed 283.07.

Synthesis of 37

A mixture of compound **36** (7.0 g, 26.9 mmol) and AcONa (22.1 g, 269.0 mmol) in water (150 mL) was refluxed for 23 h. After cooling at 0°C, the reaction mixture was acidified to pH 5 with 1N HCl. The precipitation was filtrated and dried in vacuo to give **37** (3.5 g, 54%)as a white powder .

^{1}H-NMR (400MHz, $CDCl_3$) δ 2.72 (3H, s), 6.55 (1H, s), 6.60 (1H, s), 6.68 (1H, s), 6.75-6.77 (1H, d, J = 8.8 Hz), 7.87-7.89 (1H, d, J = 8.4 Hz). ESI-TOF-MS: m/z for $C_{14}H_9O_4$: calcd 241.05 $[M-H]^-$, observed 241.05.

Synthesis of 7

To a solution of **37** (1.00 g, 4.13 mmol) and DIEA (1.72 mL, 12.39 mol) in dry CH_2Cl_2 (30 mL), was added dropwise pivaloyl chloride (1.13 mL, 9.09 mmol). The reaction mixture was stirred at rt for 1 h. After dilution with water, the residue was extracted with AcOEt. The organic layers were washed with saturated $NaHCO_3$, water and brine followed by driying over Na_2SO_4. After removal of the solvent in vacuo, the residue was washed with hexane to give **7** (1.05 g, 62%) as a white solid.

^{1}H-NMR (400MHz, $CDCl_3$) δ 1.39 (18H, s), 2.92 (3H, s), 6.86 (1H, s), 7.06-7.08 (1H, dd, J = 2.0 Hz, 8.8 Hz), 7.10 (1H, d, J = 2.0 Hz), 7.19-7.20 (1H, d, J = 2.0 Hz), 8.27-8.30 (1H, d, J = 8.8 Hz). ESI-TOF-MS: m/z for $C_{24}H_{26}NaO_6$: calcd 433.16 $[M+H]^+$, observed 433.16.

Scheme S8. Synthesis of 14

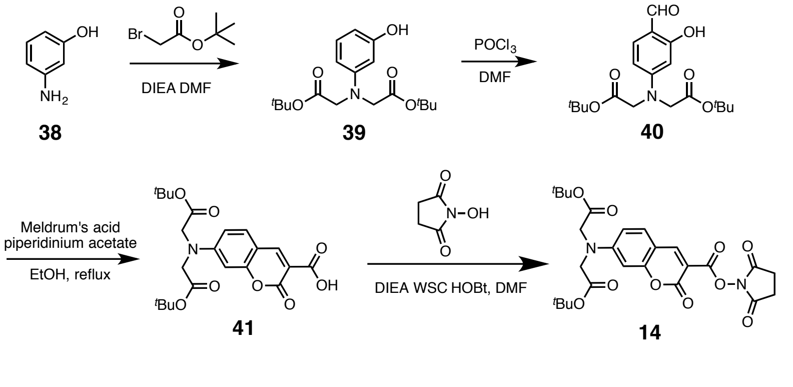

Synthesis of 39

To a solution of 3-aminophenol **38** (1.0 g, 9.17 mmol) and DIPEA (4.8 mL, 27.5 mmol) in dry DMF (30 mL) was added *t*-butylbromoacetate (2.5 mL, 18.3 mmol). The solution was stirred at 60 °C for 20 h. After dilution with $CHCl_3$, the mixture was washed with saturated $NaHCO_3$ and brine followed by drying over Na_2SO_4. The solvent was removed in vacuo and the residue was purified by flash column chromatography on silica gel (hexane / AcOEt = 10 / 1) to give **39** (1.1 g, 37%) as an orange solid.

^{1}H-NMR (400MHz, $CDCl_3$) δ 1.46 (18H, s), 3.98 (4H, s), 4.75 (1H, s), 6.08-6.09 (1H, t, J = 2.8 Hz), 6.15-6.17 (1H, dd, J = 2.4 Hz, 8.4 Hz), 6.21-6.23 (1H,dd, J = 2.0 Hz, 8.0 Hz), 7.02-7.06 (1H, t, J = 8.4 Hz). ESI-TOF-MS: *m/z* for $C_{18}H_{27}NNaO_5$: calcd 360.18 $[M+Na]^+$, observed 360.17.

Synthesis of 40

To a cooled (0 °C) solution of $POCl_3$ (440 μL, 4.67 mmol) in dry DMF (30 mL) was added dropwise a solution of **39** (1.1 g, 3.1 mmol) in dry DMF (10 mL) . The mixture was stirred at rt for 6 h and then poured into ice-cold water. After stirring for 10 min, the precipitate was filtrated, washed with water and dried in vacuo to give **40** (0.97 g, 86%) as a white powder.

^{1}H-NMR (400MHz, $CDCl_3$) δ 1.48 (18H, s), 4.05 (4H, s), 6.03-6.04 (1H, d, J = 2.4 Hz), 6.18-6.21 (1H, dd, J = 2.4 Hz, 9.2 Hz), 7.31-7.34 (1H, d, J = 9.2 Hz), 9.58 (1H, s), 11.46 (1H, s). ESI-TOF-MS: *m/z* for $C_{19}H_{27}NNaO_6$: calcd 388.17 $[M+Na]^+$, observed 388.17.

Synthesis of 41

To a solution of **40** (400 mg, 1.26 mmol), Meldrum's acid (190 mg, 1.51 mmol) and catalytic amount of piperidium acetate in dry EtOH (10 mL) was refluxed for 13 h. After cooling to rt, the precipitate was filtrated and washed with EtOH to give **41** (255 mg, 47%) as a yellow powder.

^{1}H-NMR (400MHz, $CDCl_3$) δ 1.50 (18H, s), 4.11 (4H, s), 6.51 (1H, d, J = 2.8 Hz), 6.64-6.67 (1H, dd, J = 2.8 Hz, 9.2 Hz), 7.52-7.54 (1H, d, J = 8.8 Hz), 8.74 (1H, s). ESI-TOF-MS: *m/z* for $C_{22}H_{28}NO_8$: calcd 434.18 $[M+H]^+$, observed 434.18.

Synthesis of 14

A solution of **41** (56 mg, 129 μmol), *N*-hydroxysuccinimide (18.1 mg, 155 mmol) and WSC-HCl (29.8 mg, 155 μmol) in dry DMF (3 mL) was stirred for 12 h. The mixture was diluted with water and extracted with AcOEt. The organic layere werewashed with water and brine followed by drying over Na_2SO_4. After removal of the solvent in vacuo, the residue was purified by flash column chromatography on silica gel ($CHCl_3$ / MeOH = 15 / 1) to give **14** (53 mg, 77%) as a yellow solid.

^{1}H-NMR (400MHz, $CDCl_3$) δ 1.49 (18H, s), 2.88 (4H, s), 4.10 (4H, s), 6.43-6.44 (1H, d, J = 2.4 Hz), 6.57-6.60 (1H, dd, J = 2.0 Hz, 9.2 Hz), 7.43-7.46 (1H, d, J = 8.8 Hz), 8.64 (1H, s). ESI-TOF-MS: *m/z* for $C_{26}H_{31}N_2O_{10}$: calcd 531.20 $[M+H]^+$, observed 531.19.

Scheme S9. Synthesis of 14-AM

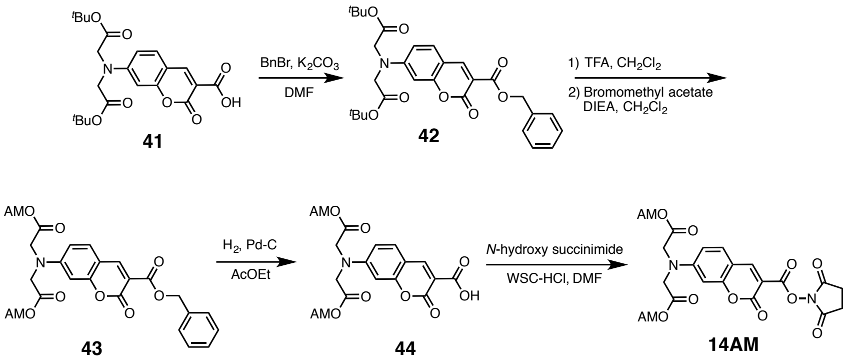

Synthesis of 42

To a solution of **41** (150 mg, 350 μmol), K_2CO_3 (176 mg, 1050 mmol) in dry DMF (3 mL) was added benzyl bromide (63 μL, 0.53 mmol). The mixture was stirred at rt for 30 min. After dilution with AcOEt, the organic layers were washed with saturated $NaHCO_3$, water and brine followe by drying over Na_2SO_4. The solvent was removed in vacuo, and the residue was purified by flash column chromatography on silica gel ($CHCl_3$) to give **42** (170 mg, 93%) as a yellow oil.

^{1}H-NMR (400MHz, $CDCl_3$) δ 1.48 (18H, s), 4.07 (4H, s), 5.36 (2H, s), 6.42-6.43 (1H, d, J = 2.4 Hz), 6.52-6.55 (1H, dd, J = 2.8 Hz, 8.8 Hz), 7.33-7.40 (4H, m), 7.46-7.48 (2H, d, J = 7.6 Hz), 8.45 (1H, s). ESI-TOF-MS: *m/z* for $C_{29}H_{33}NNaO_8$: calcd 546.21 $[M+H]^+$, observed 546.21.

Synthesis of 43

To a solution of **42** (170 mg, 325 μmol) in dry CH_2Cl_2 (2 mL) was added trifluoroacetic acid (2 mL). The mixture was stirred at rt for 1 h and then concentrated in vacuo. After co-evaporated with $CHCl_3$ (×2), the residue dissolved in dry CH_2Cl_2 (2 mL) was added DIEA (254 μL, 1330 μmol) and bromomethyl acetate (150 μL, 1330 μmol). The mixture was stirred at rt for 19 h. After dilution with AcOEt., the organic layer was washed with saturated $NaHCO_3$, water and brine followed by drying over Na_2SO_4. The solvent was evaporated in vacuo, and the residue was purified by flash column chromatography on silica gel ($CHCl_3$ / MeOH = 30 / 1) to give **43** (210 mg, quant, as mono bromide salt) as a yellow oil.

^{1}H-NMR (400MHz, $CDCl_3$) δ 2.13 (6H, s), 4.26 (4H, s), 5.37 (2H, s), 5.82 (4H, s), 6.44 (1H, d, J = 2.4 Hz), 6.55-6.58 (1H, dd, J = 2.4 Hz, 8.8 Hz), 7.32-7.48 (6H, m), 8.46 (1H, s). ESI-TOF-MS: *m/z* for $C_{27}H_{25}NNaO_{12}$: calcd 578.13 $[M+H]^+$, observed 578.13.

Synthesis of 44

To a solution of compound **43** (210 mg, 325 μmol) in AcOEt (4 mL) was added 10% Pd/C (35 mg) under N_2 atmosphere. The N_2 gas was degassed and backfilled with H_2 gas twice. The solution was stirred at rt for 24 h. After removal of Pd/C by filtration, the solvent was concentrated in vacuo. The residue was purified by flash column chromatography on silica gel ($CHCl_3$ / MeOH = 30 / 1) to give **44** (55 mg, 36%) as a yellow powder.

^{1}H-NMR (400MHz, DMSO-d6) δ 2.08 (6H, s), 4.49 (3H, s), 5.73 (3H, s), 6.60 (1H, s), 6.72-6.75 (1H, d, J = 10.0 Hz), 7.68-7.70 (1H, d, J = 8.8 Hz), 8.62 (1H, s). ESI-TOF-MS: *m/z* for $C_{20}H_{18}NO_{12}$: calcd 464.08 $[M-H]^-$, observed 464.08.

Synthesis of **14-AM**

To a solution of **44** (55 mg, 118 μmol), *N*-hydroxy succinimide (17.0 mg, 142 mmol) and WSC-HCl (27.0 mg, 142 μmol) in dry DMF (5 mL) was stirred for 4 h. The mixture was diluted with AcOEt and the organic layers were washed with saturated $NaHCO_3$, water and brine and dried over Na_2SO_4. After removal of the solvent in vacuo, the residue was purified by flash column chromatography on silica gel ($CHCl_3$ / MeOH = 30 / 1) to give **14-AM** (24 mg, 36%) as a yellow oil.

^{1}H-NMR (400MHz, $CDCl_3$) δ 2.14 (6H, s), 2.88 (4H, s), 4.28 (4H, s), 5.83 (4H, s), 6.45-6.46 (1H, d, J = 2.4 Hz), 6.60-6.63 (1H, dd, J = 2.4 Hz, 8.8 Hz), 7.47-7.50 (1H, d, J = 8.8 Hz), 8.66 (1H, s). ESI-TOF-MS: *m/z* for $C_{24}H_{23}N_2O_{14}$: calcd 563.11 $[M+H]^+$, observed 563.11.

Scheme S10. Synthesis of 46

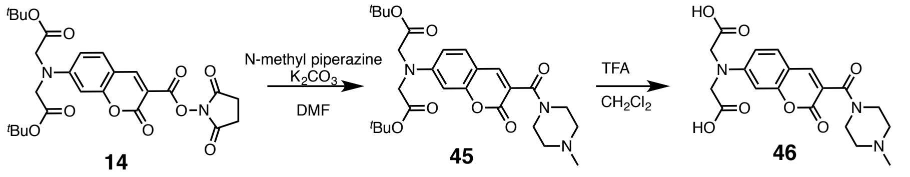

Synthesis of 45

To a solution of **14** (30 mg, 57 μmol), DIEA (48 μL, 280 μmol) in dry DMF (3 mL) was added methyl piperazine (17 μL, 170 μmol). The reaction mixture was stirred at rt for 10 h. After dilution with

water, the mixture was extracted with AcOEt. The organic layers were washed with saturated $NaHCO_3$, water and brine. The solvent was dried over Na_2SO_4 and concentrated in vacuo to give **45** (21 mg, 73%) as a yellow solid.

^{1}H-NMR (400MHz, $CDCl_3$) δ 1.48 (18H, s), 2.34 (3H, s), 2.48 (4H, brs), 3.43 (2H, brs), 3.79 (2H, brs), 4.06 (4H, s), 6.44 (1H, s), 6.53-6.55 (1H, d, J = 8.8 Hz), 7.33-7.36 (1H, d, J = 8.4 Hz), 7.83 (1H, s). ESI-TOF-MS: *m/z* for $C_{27}H_{38}N_3O_7$: calcd 516.27 $[M+H]^+$, observed 516.26.

Synthesis of 46

To a solution of **45** (21 mg, 42 μmol) in CH_2Cl_2 (2 mL) was added trifluoroacetic acid (2 mL). The reaction mixture was stirred at rt for 5 h. The solvent was evaporated in vacuo to give **46** (20 mg, 92% as mono TFA salt) as a yellow solid.

^{1}H-NMR (400MHz, CD_3OD) δ 2.97 (3H, s), 3,47 (8H, br), 4.32 (4H, s), 6.55 (1H, s), 6.72-6.74 (1H, d, J = 8.8 Hz), 7.54-7.56 (1H, d, J = 8.8 Hz), 8.08 (1H, s). ESI-TOF-MS: *m/z* for $C_{19}H_{21}N_3NaO_7$: calcd 426.1272 $[M+Na]^+$, observed 426.1299.

Reference

S1. I. Takashima, R. Kawagoe, I. Hamachi and A. Ojida, *Chem. Eur. J.*, 2014, **21**, 2038–2044.
